# The highly conserved FOXJ1 target CFAP161 is dispensable for motile ciliary function in mouse and *Xenopus*

**DOI:** 10.1038/s41598-021-92495-3

**Published:** 2021-06-25

**Authors:** Anja Beckers, Franziska Fuhl, Tim Ott, Karsten Boldt, Magdalena Maria Brislinger, Peter Walentek, Karin Schuster-Gossler, Jan Hegermann, Leonie Alten, Elisabeth Kremmer, Adina Przykopanski, Katrin Serth, Marius Ueffing, Martin Blum, Achim Gossler

**Affiliations:** 1 Institute for Molecular Biology, OE5250, Hannover Medical School, Carl-Neuberg-Str. 1, 30625 Hannover, Germany; 2 Institute of Biology, University of Hohenheim, Garbenstraße 30, 70593 Stuttgart, Germany; 3 Institute of Ophthalmic Research, Center for Ophthalmology, University of Tübingen, Elfriede-Aulhorn-Strasse 7, 72076 Tübingen, Germany; 4 Renal Division, Department of Medicine, University Hospital Freiburg, Freiburg University Faculty of Medicine & CIBSS-Centre for Integrative Biological Signaling Studies, University of Freiburg, Habsburger Str. 49, 79104 Freiburg, Germany; 5 Institute of Functional and Applied Anatomy, Research Core Unit Electron Microscopy, OE8840, Hannover Medical School, Carl-Neuberg-Str. 1, 30625 Hannover, Germany; 6 Institute of Molecular Immunology, Helmholtz Zentrum München, German Research Center for Environmental Health (GmbH), Core Facility Monoclonal Antibodies, Marchioninistr. 25, 81377 München, Germany; 7 Present Address: Renal Division, Department of Medicine, University Hospital Freiburg, Freiburg University Faculty of Medicine & CIBSS-Centre for Integrative Biological Signalling Studies, University of Freiburg, Habsburger Str. 49, 79104 Freiburg, Germany; 8 Present Address: Twist Bioscience, 681 Gateway Blvd South, South San Francisco, CA 94080 USA; 9 Present Address: Department of Biology II, Ludwig-Maximilians University, Großhaderner Straße 2, 82152 Martinsried, Germany; 10 Present Address: Institute for Toxicology, OE 5340, Hannover Medical School, Carl-Neuberg-Str. 1, 30625 Hannover, Germany

**Keywords:** Cell biology, Model vertebrates, Ciliogenesis

## Abstract

Cilia are protrusions of the cell surface and composed of hundreds of proteins many of which are evolutionary and functionally well conserved. In cells assembling motile cilia the expression of numerous ciliary components is under the control of the transcription factor FOXJ1. Here, we analyse the evolutionary conserved FOXJ1 target CFAP161 in *Xenopus* and mouse. In both species *Cfap161* expression correlates with the presence of motile cilia and depends on FOXJ1. Tagged CFAP161 localises to the basal bodies of multiciliated cells of the *Xenopus* larval epidermis, and in mice CFAP161 protein localises to the axoneme. Surprisingly, disruption of the *Cfap161* gene in both species did not lead to motile cilia-related phenotypes, which contrasts with the conserved expression in cells carrying motile cilia and high sequence conservation. In mice mutation of *Cfap161* stabilised the mutant mRNA making genetic compensation triggered by mRNA decay unlikely. However, genes related to microtubules and cilia, microtubule motor activity and inner dyneins were dysregulated, which might buffer the *Cfap161* mutation.

## Introduction

Cilia are extensions that protrude from the cell surface of most vertebrate and some invertebrate cell types into the extracellular space. They can be broadly grouped into nonmotile and motile cilia^1^. Nonmotile cilia, also referred to as primary cilia, are present on virtually every cell of vertebrates and are critical for signal transduction and sensing of external stimuli (reviewed in^2,3^). Motile cilia move extracellular fluids along epithelia, or propel cells through the surrounding medium (e.g.^4–6^).

Rotational movement of single motile cilia on cells of the left–right organiser of vertebrate embryos generates a leftward fluid flow, which is pivotal for establishing the asymmetric arrangement of visceral organs^7–9^. Coordinated whip-like beating of multiple cilia on ependymal cells of the brain, on epithelial cells of the respiratory tract, or of the fallopian tube are essential for cerebrospinal fluid flow^6,10–12^, mucociliary clearance^5,13^, and movement of eggs into the ampulla and towards the uterus^14^, respectively. Wave-like movement of the sperm flagellum, a specialised long cilium, is essential for sperm motility and fertilisation^4^.

Defects in the formation of cilia or their function cause human diseases collectively known as ciliopathies (reviewed in^15–19^). A specific subgroup of ciliopathies, referred to as primary cilia dyskinesia (PCD), is caused by impaired function of motile cilia. Reflecting the functions of motile cilia, PCD is characterised by situs randomisation, impaired mucociliary clearance, respiratory problems, and male infertility. Mouse models of PCD frequently show reduced female fertility and hydrocephalus^12^, which are less common in human patients (reviewed in^20,21^).

The formation of apparently all motile cilia in the mouse as well as in other vertebrates and invertebrates is under the control of the transcription factor FOXJ1^22–28^. Thus, genes that act downstream of FOXJ1 are likely to be required for the formation or function of motile cilia. In microarray screens for FOXJ1 target genes in node-stage embryos and foetal lungs^29^ we have identified a number of new *Foxj1* targets^30–34^ including *Cfap161*, the orthologue of the axonemal *Chlamydomonas reinhardtii* FAP161 protein^35^. Recently, single-particle cryo-electron microscopy of *Chlamydomonas* flagella showed that FAP161 is one of the 33 microtubule inner proteins (MIPs) and likely localises to the A-tubule of the outer doublets^36^. Morpholino-mediated knock down of *Cfap161* in zebrafish led to the loss of outer dynein arms, reduced beating frequency of pronephric cilia and strong ciliopathy phenotypes including left–right asymmetry defects in one study^37^, but only a curved body axis and hydrocephalus in a subsequent analysis^38^ leaving some uncertainties concerning the ciliary function of CFAP161. Human *CFAP161* (c15orf26) is located on chromosome 15q in the linkage region of Kartagener syndrome^39^, suggesting a potential involvement of *Cfap161* mutations in this subtype of PCD. Here, we describe the analysis of CFAP161 function in *Xenopus* crispants and mutant mice generated by homologous recombination. Surprisingly, disruption of the highly conserved *Cfap161* gene did not lead to obvious phenotypes related to dysfunctional motile cilia.

## Results

### Expression of *Cfap161* in mouse and *Xenopus*

**Figure 1:**
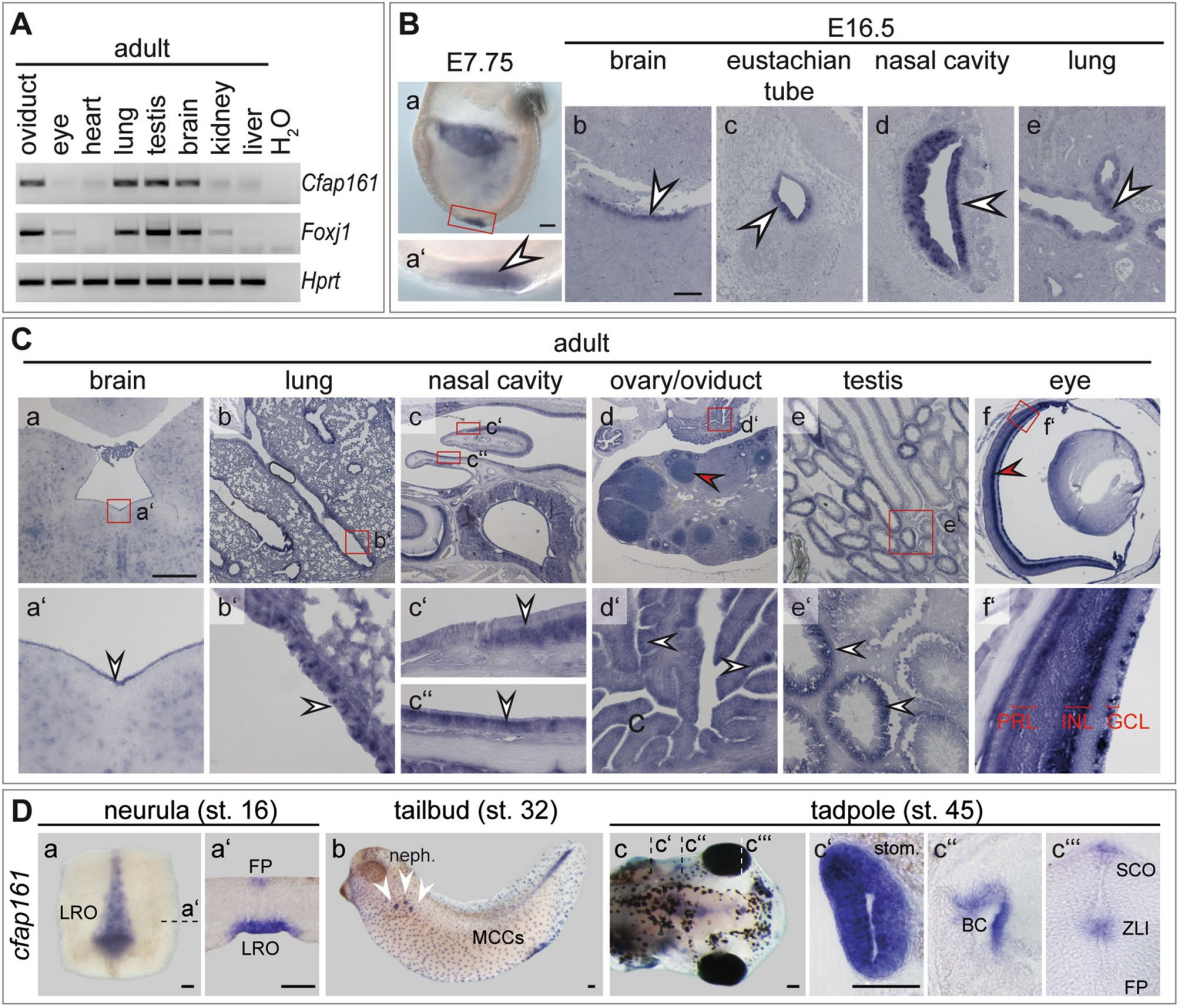
Expression of *Cfap161* in mouse and *Xenopus*. (**A**) Analysis of *Cfap161* and *Foxj1* expression by RT-PCR of RNA from wild type adult organs as indicated on top. *Hprt* was used as quality control. The full-size agarose gel is shown in Fig. S8. (**B**) WISH of E7.75 wild type murine embryo (a, a′) and SISH of E16.5 wild type embryos showing *Cfap161* expression (arrowheads) in the node (a) and indicated tissues (b–e) that develop motile cilia. Red boxed area in (a) indicate the region shown in higher magnification in (a′). (**C**) SISH analysis of wild type adult tissues (indicated on top). Red boxed areas in (a–f) indicate the regions shown at higher magnification in (a′–f′). White arrowheads point to regions of expression in cells possessing motile cilia. Red arrowheads point to regions of cells with primary cilia. *PRL* photoreceptor layer, *INL* inner nuclear layer, *GCL* ganglion cell layer. (**D**) *Xenopus* WISH detected *cfap161* transcripts in the left–right organiser (LRO; a, a′), floor plate (FP; a′), nephrostomes marked by white arrowheads (b), multiciliated cells (MCCs) of the skin (b), stomach (stom.; c, c′), branchial chamber (BC; c, c″) subcommissural organ (SCO; c, c‴) and in the zona limitans intrathalamica (ZLI; c, c‴). Transversal section planes are indicated with stippled lines and shown in (a′, c′, c″, c‴). Scale bars: (**B**, **D**) = 100 µm; (**C**) = 500 µm.

Mouse *Cfap161* (RefSeq NM_029335.3) encodes an evolutionary conserved (Table S1) 303 amino acids (aa) protein lacking domains of known biochemical function. High expression levels of *Cfap161* correlate in general with the presence of motile cilia and co-expression of *Foxj1* (Fig. 1A). In early embryos (E7.75) *Cfap161* is expressed in the ciliated ventral layer of the embryonic node (the mouse left–right organiser, LRO; Fig. 1Ba,a′), and in later foetal stages (E16.5) it is expressed in ependymal cells of the brain (Fig. 1Bb), epithelial cells lining the eustachian tube (Fig. 1Bc), the respiratory epithelium of the nasal cavity (Fig. 1Bd), and in ciliated epithelial cells of the lung (Fig. 1Be). In these tissues expression was also detected in adults (Fig. 1A) as well as in cells of the oviduct carrying motile cilia, and in testis (Fig. 1A,Ca–e). In testis, *Cfap161* transcripts were not found in all seminiferous tubules (Fig. 1Ce,e′) suggesting that *Cfap161* transcription is restricted to distinct stages of the cycle of the epithelium of the seminiferous tubules. Consistent with this idea, CFAP161 protein was only observed at late stages of spermiogenesis (Fig. S1). *Cfap161* mRNA was additionally detected in some cells lacking motile cilia: in the retina, in the ganglion cell layer (GCL), the inner nuclear layer (INL), in the photoreceptor cells (PRL; Fig. 1Cf, red arrowhead in f′) and in developing follicles (red arrowhead in Fig. 1Cd). Likewise, CFAP161 protein was detected in cells harbouring immotile cilia (primary cilia), such as hair cells in the inner ear or in cells of the kidney collecting ducts (Fig. S2).

Consistent with the prominent expression in cells carrying motile cilia in mouse tissues, *cfap161* was detected in the left–right organiser (LRO; Fig. 1Da,a′) and in the floor plate of the prospective neural tube (Fig. 1Da′) during neurulation in *Xenopus laevis* embryos. Tailbud stages showed transcripts in multiciliated cells (MCCs) of the epidermis and in the nephrostomes (Fig. 1Db). Free-swimming tadpoles established expression domains in the stomach (stom.; Fig. 1Dc,c′), branchial chamber (BC; Fig. 1Dc,c″), subcommissural organ (SCO) and in the zona limitans intrathalamica (ZLI; Fig. 1Dc,c‴).

### Localisation of CFAP161 and dependence on FOXJ1

**Figure 2:**
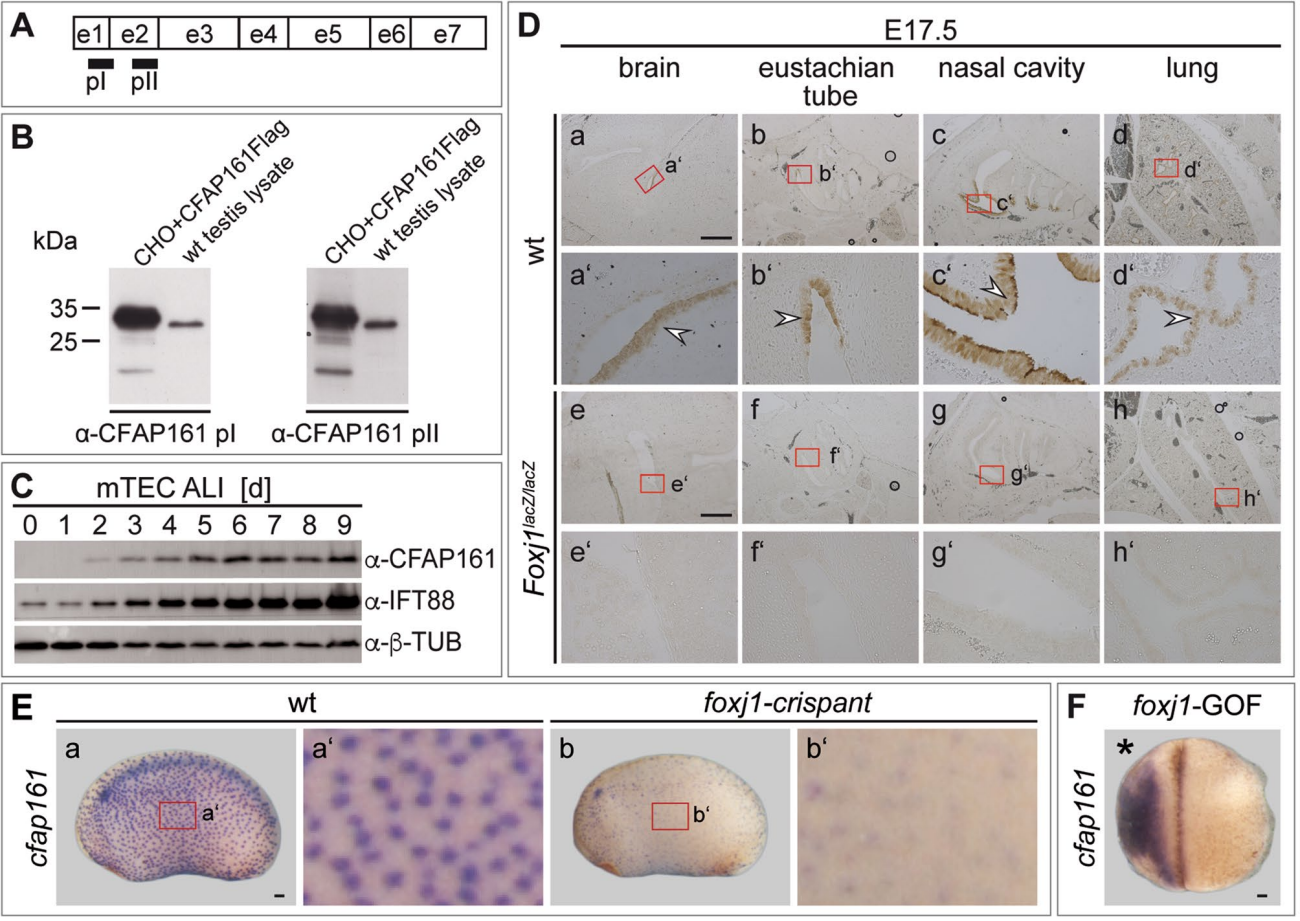
Localisation of CFAP161 and the dependence on FOXJ1. (**A**) Schematic representation of the exon structure of *Cfap161* and regions encoding the peptides (pI and pII) used for the generation of antibodies. (**B**) Western blot of overexpressed (CFAP161Flag in CHO cells) and endogenous CFAP161 (from mouse testis lysate) detected with monoclonal (pI) and polyclonal (pII) antibodies. The full-size Western blots are shown in Fig. S9A. (**C**) Induction of CFAP161 expression during cilia formation of air–liquid interface (ALI) cultures of mouse tracheal epithelial cells (mTEC). α-CFAP161 pII was used for visualising CFAP161, α-IFT88 to monitor ciliogenesis and α-β-Tubulin (β-TUB) as loading control. The full-size Western blot is shown in Fig. S9B. (**D**) Detection of endogenous CFAP161 by indirect immunohistochemistry of E17.5 wild type and *Foxj1*-mutant sections. Red boxed areas in a-h indicate the regions shown at higher magnification in a′–h′. Note that CFAP161 is absent in all analysed tissues of *Foxj1*^lacZ/lacZ^ specimens. α-CFAP161 pI was used for the indirect DAB-immunostaining. (**E**) Expression of *cfap161* was largely erased in *foxj1*-crispant embryos (b, b′) in comparison to wild types (a, a′). Red boxed areas in (a, b) indicate the regions shown at higher magnification in (a′, b′). (**F**) Unilateral *foxj1* gain of function (*foxj1*-GOF) induced *cfap161*. Side of injection as indicated by asterisk. Scale bars: (**D**) = 500 µm; (**E**, **F**) = 100 µm.

To study CFAP161 protein expression in mouse we generated a monoclonal rat antibody directed against a peptide (α-pI) mainly encoded by exon 1 (Fig. 2A and Fig. S3) and polyclonal antibodies directed against a peptide (α-pII) encoded by exon 2 in rabbits (Fig. 2A). In Western blots both antibodies detected Flag-tagged CFAP161 over-expressed in CHO cells as well as endogenous CFAP161 in testis lysates (Fig. 2B). Additionally, these antibodies detected CFAP161 expression concomitantly with cilia formation indicated by co-expression with the ciliary protein IFT88 in air–liquid interface (ALI) cultures of mouse tracheal epithelial cells (mTECs) (Fig. 2C). The monoclonal antibody detected endogenous CFAP161 in foetal (E17.5) tissue sections (Fig. 2D), consistent with *Cfap161* mRNA expression domains (Fig. 1Bb–e) corroborating the specific expression of *Cfap161* in cells carrying motile cilia. CFAP161 was not detected in tissues of *Foxj1* null mutant (*Foxj1*^*lacZ/lacZ*^) foetuses (n = 4; Fig. 2De–h). Likewise, *Xenopus foxj1*-crispants revealed a global downregulation of *cfap161* as prominently seen in the epidermis (Fig. 2Eb,b′) where expression of *cfap161* is nearly absent in the MCCs, and a unilateral *foxj1* gain of function (*foxj1*-GOF) strongly induced transcription of *cfap161* (Fig. 2F) confirming the dependence of *Cfap161* expression on FOXJ1 as was previously observed in zebrafish^37^.

**Figure 3:**
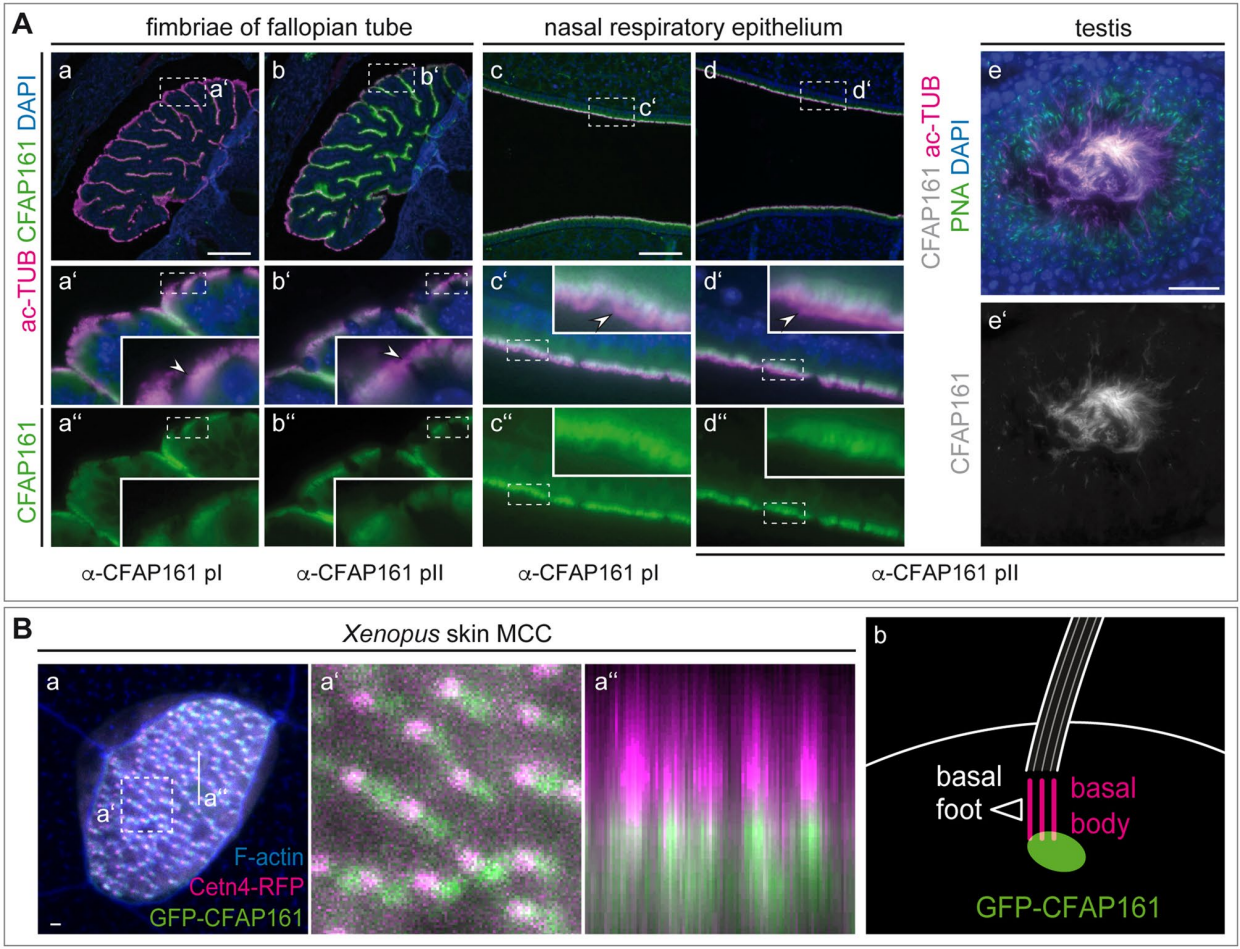
Subcellular localisation of CFAP161. (**A**) Indirect immunofluorenscence staining of murine adult fallopian tube sections (a, b), sections of adult respiratory epithelium of the nasal cavity (c, d), and seminiferous tubule of the adult testis (e, e′) showing localisation of CFAP161 to the ciliary axoneme and flagella, respectively. The distal region of cilia marked by white arrowhead lacks CFAP161. Boxed areas in a-d indicate the regions shown at higher magnification in a′–d′ and a″–d″, boxed areas in a′–d′ and a″–d″ indicate the regions shown enlarged in the respective insets. (**B**) Localisation of mouse GPF-CFAP161 in *Xenopus* MCCs co-expressing the basal body marker Cetn4-RFP (a) revealed accumulation of CFAP161 in anterior juxtaposition (a′) underneath Cetn4 (a″) as illustrated in (b). Boxed area in (a) indicate the region shown at higher magnification in (a′). Orthogonal section as indicated and shown in (a″). Scale bars: (**A**)a–d = 100 µm; (**A**)e, e′ = 25 µm; B = 100 µm.

Indirect immunofluorescence of adult tissue sections using α-pI and α-pII antibodies showed co-localisation of CFAP161 with acetylated-α-tubulin (ac-TUB; Fig. 3A), indicating that CFAP161 is a component of motile cilia. CFAP161 and ac-TUB staining did not overlap in the distal part of cilia (Fig. 3Aa′–d′, arrowheads in insets) indicating that CFAP161 is excluded from the tip of the cilium, which was most clearly seen in the sections of the nasal respiratory epithelium (Fig. 3Ac′,d′). Absence of CFAP161 from the cilium tip is consistent with the localisation of its *Chlamydomonas* orthologue in outer microtubule doublets and likely reflects the fact that outer doublets terminate earlier than the central inner pair^40^. Consistent with the ciliary localisation, CFAP161 was found in the flagellum of spermatozoa (Fig. 3Ae,e′; Fig. S1). Detection of *Xenopus* CFAP161 failed with α-pI and α-pII antibodies but heterologously expressed murine CFAP161 tagged with a N-terminal EGFP (GFP-CFAP161) partially co-localised with a Cetn4-RFP fusion construct, which marks the basal bodies of epidermal MCCs (Fig. 3Ba). Of note, most GFP-CFAP161 signal was in anterior juxtaposition underneath each basal body (Fig. 3Ba′,a″), reminiscent of the rootlet (Fig. 3Bb). Localisation at basal bodies might reflect non-physiological accumulation due to high levels of the overexpressed GFP-tagged CFAP161. Similarly, also overexpressed N- or C-terminally HA-tagged *Xenopus* Cfap161 was not specifically localised in cilia but found throughout the whole cell (Fig. S4). It appears unlikely that in *Xenopus* this conserved ciliary protein is absent from motile cilia. Rather, the apparent uniform distribution of HA-tagged Cfap161 might reflect high levels of overexpressed HA-tagged Cfap161 that exceed the amount that can translocate to cilia.

To obtain first insights into the biochemical context in which CFAP161 might act, we identified potential interaction partners by mass spectrometry of CFAP161 complexes immunoprecipitated from wild type testis lysates using the polyclonal α-pII antibody (Table S2). One potential interaction partner was KIAA0556 (also known as Katanin-interacting protein KATNIP), a basal body protein that stabilises cytoplasmic microtubules in human cells, regulates ciliary A-tubule number in *C. elegans,* and when mutated, causes Joubert syndrome in humans^41^. Yeast-two-hybrid analysis (Fig. S5) validated a robust interaction of CFAP161 and KIAA0556, supporting a function of CFAP161 in some aspects of microtubule organisation or function.

### Functional analyses of CFAP161 in *Xenopus*

**Figure 4:**
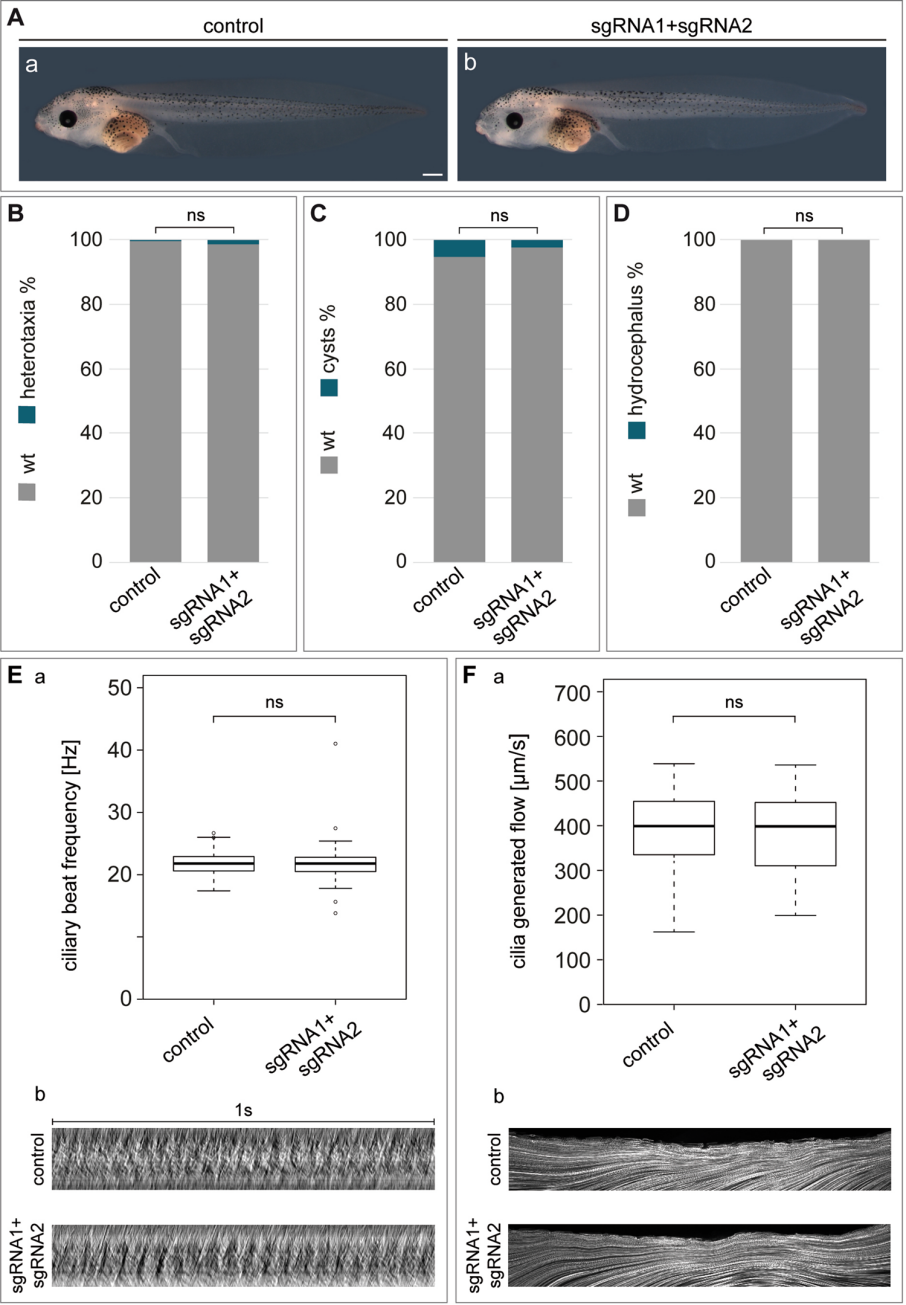
No cilia related phenotypes in *Xenopus cfap161*-crispants. (**A**) Representative wild type (a) and crispant (b) tadpoles at stage 45. (**B**) Evaluation of organ situs, embryonic cysts (**C**) and hydrocephalus (**D**) in 297 crispants obtained in 5 independent experiments. Statistical calculation of organ situs defects, cyst analysis or hydrocephalus formation was performed using chi square (http://www.physics.csbsju.edu/stats/contingency_NROW_NCOLUMN_form.html). (**E**) Ciliary beat frequency (CBF) in wild type and crispants. (a) Statistical evaluation of CBF. Results from 3 independent experiments with each 5 embryos and 5 analysed MCCs per embryo. Graph displays respective values with mean and s.d. Raw data are shown in Table S3. (b) Kymographs of ciliary motility of single MCCs of wild type and crispant embryos. (**F**) Cilia generated flow (CGF) (a) Statistical evaluation of CGF, velocities of bead transport in wild type and crispant embryos. Results from 3 independent experiments with 8 analysed specimens each. Graph displays respective values with mean and s.d. Raw data are shown in Table S4. (b) Maximum intensity projection of bead transport of single wild type and crispant embryo. Compared to wild type specimens no significant deviations were recorded in any experiment. ns P > 0.05. p-values were calculated via Wilcoxon-Match-Pair test in RStudio. Scale bar: (**A**) = 500 µm.

To investigate the function of CFAP161 in the frog *Xenopus* we mutated *cfap161* by genome editing using CRISPR/Cas9. Two different single guide RNAs (sgRNAs) were designed, targeting exon 1 (sgRNA1) and exon 3 (sgRNA2), respectively. Sequencing of PCR products with pooled DNAs of 10 F0 crispants (Fig. S6A,B) confirmed successful genome editing of *cfap161*. Furthermore, *cfap161* transcripts were strongly reduced in *cfap161*-crispants, indicative for nonsense mediated mRNA decay (Fig. S6Cb,b′). Initial experiments with individual sgRNAs showed no effect in ciliated cells of the larval epidermis (data not shown). Subsequently both sgRNAs (sgRNA1 & sgRNA2) were injected simultaneously. Crispants were analysed for laterality defects, the development of pronephric cysts or an externally visible hydrocephalus in stage 45 tadpoles. Evaluation of 297 crispants obtained in five independent experiments showed no impact on their overall phenotype (Fig. 4A–D). To address whether loss of *cfap161* has a more subtle effect on ciliary beating of epidermal MCCs, we analysed high speed video microscopy recordings of stage 32 wild type and crispant specimens for ciliary beat frequency (CBF) and cilia generated flow (CGF) as described in detail in^33^. Neither CBF (approximately 22 Hz in both wild type and crispant embyos; Fig. 4Ea,b; Table S3) nor CGF (around 400 µm/s for both wild type and crispant embryos; Fig. 4Fa,b; Table S4) was affected by mutating *cfap161*. Thus, disruption of *cfap161* showed no phenotypic changes associated with dysfunctional motile cilia in *Xenopus*.

### Functional analysis of CFAP161 in mice

**Figure 5:**
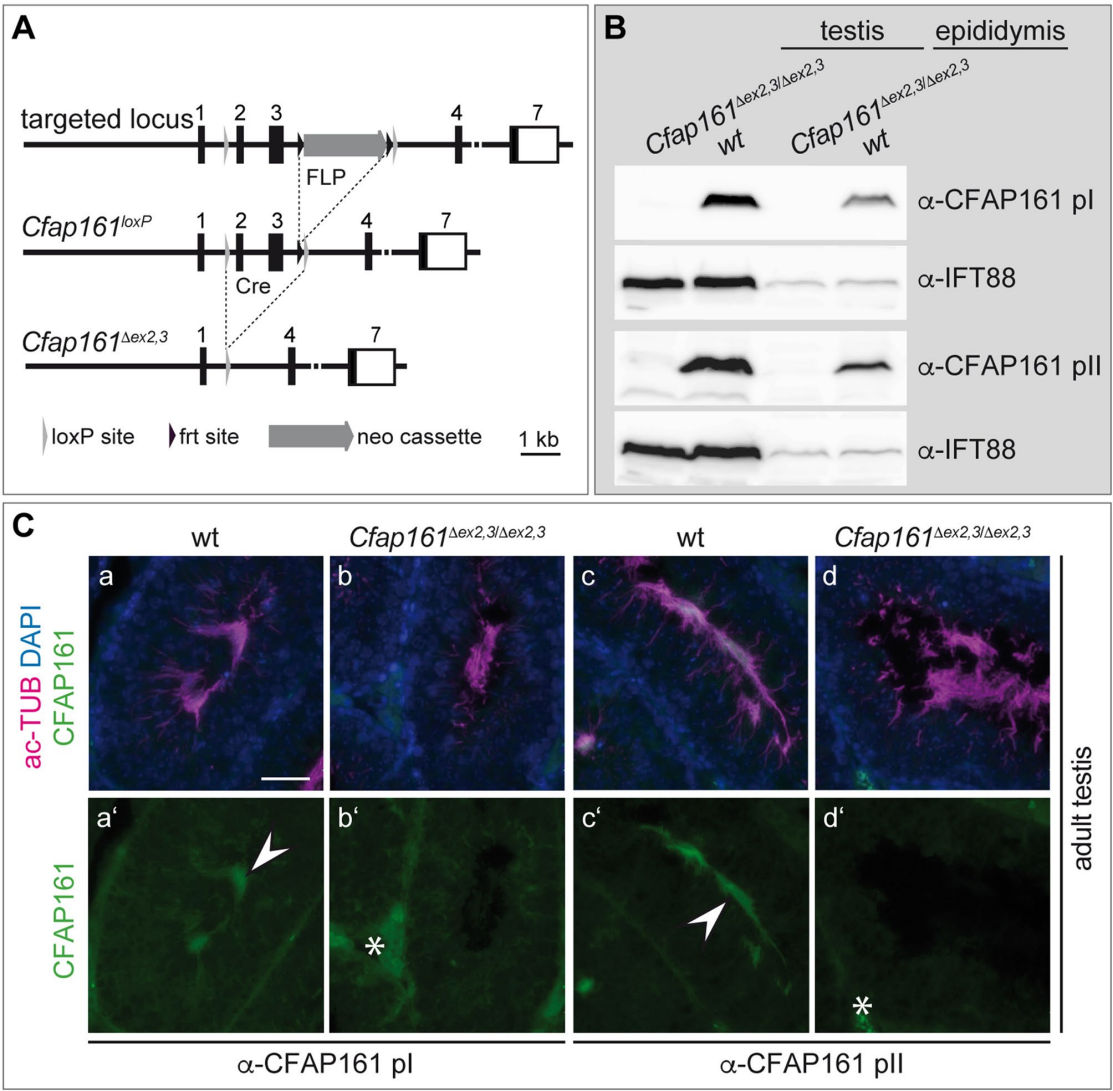
Generation of a murine *Cfap161*-null allele *Cfap161*^*∆ex2,3*^. (**A**) Schematic drawing showing the structure of the targeted *Cfap161* locus and mutated alleles (*Cfap161*^*loxP*^ and *Cfap161*^*∆ex2,3*^). (**B**) Western blots of testis and epididymis lysates with monoclonal pI and polyclonal pII antibody show absence of CFAP161 in the mutant (*Cfap161*^*∆ex2,3/∆ex2,3*^). α-IFT88 was used as loading control. The full-size Western blots are shown in Fig. S9C. (**C**) Indirect immunofluorescence of wild type (a, a′, c, c′) and *Cfap161*^*∆ex2,3*^ mutant (b, b′, d, d′) testis sections showing absence of CFAP161 from flagella of mutant sperm. White arrowheads: flagella; asterisk: non-specific staining around seminiferous tubules. Scale bar: (**C**) = 50 µm.

To analyse CFAP161 function under physiological conditions in mice we generated a conditional allele by flanking *Cfap161* exons 2 and 3 by loxP recombination sites (Fig. 5A). Deletion of these two exons by Cre-mediated site-specific recombination results in a frame shift that generates a premature translational stop codon by the first base triplet of exon 4, which should effectively abolish the generation of full-length CFAP161 protein. To generate mice lacking CFAP161 in all tissues we deleted exons 2 and 3 (*Cfap161*^*∆ex2,3*^) in the female germ line using ZP3:Cre mice^42^. Homozygous *Cfap161*^*∆ex2,3*^ mice were born at Mendelian ratio (wt 95, het 186, hom 91; χ^2^ = 0.086, p = 0.98) and showed no obvious abnormalities. Western blot analyses of testis and epididymis lysates with the monoclonal antibody α-pI (which recognises the epitope encoded by exon 1; analysis shown in Fig. S3) and with polyclonal antibody α-pII did not detect full length CFAP161 protein or any other shorter protein product (Fig. 5B; Fig. S9C) that might have been generated by alternative splicing. Likewise, our antibodies did not detect CFAP161 protein in mutant testis sections (Fig. 5Cb,d) supporting that deletion of exons 2 and 3 abolishes production of CFAP161 and thus leads to a functional null allele.

**Figure 6:**
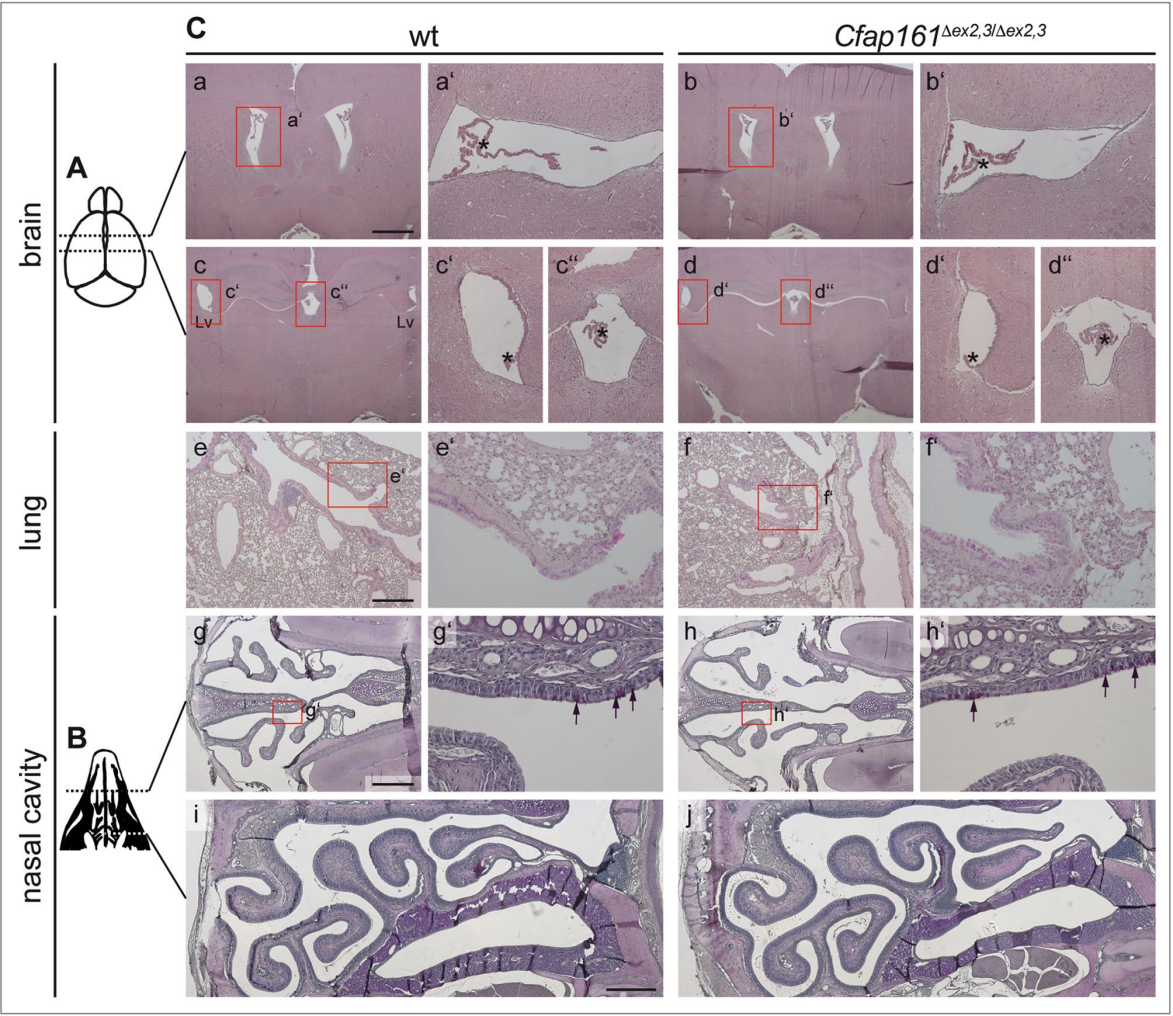
Absence of phenotypes in *Cfap161*^*∆ex2,3*^ mutants. (**A**) Schematic drawing of the brain indicating the planes of the sections shown in (**C**a–d). (**B**) Schematic drawing of the face skull indicating the planes of the sections shown in (**C**g–j). (**C**) (a–d) Sections of wild type (a, c) and *Cfap161*^*∆ex2,3*^ mutant (b, d) brains at the two horizontal levels indicated in (**A**). Red boxed areas indicate the regions shown at higher magnification in a′, b′, c′, c″, d′, and d″. Asterisks marks the choroid plexus in the ventricle lumen. (e, f) Representative sections of wild type (e) and *Cfap161*^*∆ex2,3*^ mutant (f) lungs. Red boxed areas indicate the regions shown at higher magnification in e′, and f′. Arrows point to PAS-positive (purple stained) cells, representing the mucus producing cells. (g–j) Sections of wild type (g, i) and *Cfap161*^*∆ex2,3*^ mutant (h, j) nasal cavities at the two horizontal levels indicated in (**B**). Red boxed areas indicate the regions shown at higher magnification in g′, and h′. Scale bars: (**C**)a–d: 1 mm; (**C**)e–j = 500 µm.

Disruption of normal motile cilia function can lead to hydrocephalus and mucus accumulation in the respiratory tract, abnormal *situs* of visceral organs and male infertility due to immotile spermatozoa, rarely female infertility^20^. Homozygous mutants showed no externally visible abnormalities over an observation period of ≥ 8 months, and matings of homozygous breeding pairs (n = 3) gave rise to litters with normal sizes. Serially sectioned brains (section plane is shown in Fig. 6A) of 4 months old *Cfap161*^*∆ex2,3*^ homozygotes (n = 4) did not show signs of enlarged ventricles (Fig. 6Cb,b′,d,d′,d″). Likewise, sections of mutant lungs (n = 4; Fig. 6Cf,f′) or PAS stained serial sections (section planes are shown in Fig. 6B) of the mutant nasal cavities (Fig. 6Ch,h′,j) did not show obvious morphological alterations or accumulation of mucus. Although mutant males bred apparently normal computer-assisted sperm analysis (CASA) was performed to detect potential abnormalities in sperm number or motility but revealed no differences between wild type and mutant (Fig. S7A; Table S5) samples. Likewise, electron microscopic analyses of mutant sperm cells prepared from cauda epididymis showed no obvious structural abnormalities (Fig. S7B) in the axonemes of flagella and cilia of the lung (Fig. S7C). Additionally, no polydactyly or deafness as a sign for impairment of primary cilia were observed in the mutants (n = 31). Taken together, our mutational analyses in *Xenopus* and mice did not reveal any evidence for essential CFAP161 functions in motile cilia in either vertebrate species.

### RNA-seq on CFAP161-deficient testis reveals dysregulation of ciliary gene expression

Given the unexpected result that the mutation of an evolutionary highly conserved FOXJ1 target gene has no motile cilia-related phenotype, we analysed transcriptional changes in control wild type and *Cfap161*^*∆ex2,3/∆ex2,3*^ testes by mRNA-sequencing (RNA-seq). Differential expression analysis using DEseq2 revealed a number of statistically significantly dysregulated transcripts (338; P-adj < 0.05) (Table S6). Gene-ontology (GO) analysis^43,44^ of these transcripts showed a significant enrichment of biological process terms relating to microtubule and cilia, including mitotic spindle organisation, protein localisation to cytoskeleton, microtubule bundle formation, cilium movement, and cytoskeleton-dependent intracellular transport (Table S7). Additionally, terms related to RNA biology (e.g. mRNA stabilisation, regulation of RNA processing, regulation of RNA splicing) as well as to chromatin regulation (e.g. chromatin remodelling, DNA packaging, and histone modification) were enriched. The most enriched GO-terms were ATP-dependent microtubule motor activity (minus-end-directed) and inner dynein arm when the dataset was compared to GO molecular function and GO cellular component datasets, respectively. Of the 338 dysregulated genes, 164 were downregulated and 174 were upregulated, but only few transcripts showed strong changes in expression levels (3 transcripts showed downregulation to levels of 0.5-fold-expression or smaller, and 6 showed upregulation of 2-fold-expression or higher, including *Cfap161*) (Table S8).

## Discussion

In this analysis we show that strong *Cfap161* expression correlates well with the presence of motile cilia, consistent with its dependence on FOXJ1 both in *Xenopus* and mice. In mice CFAP161 protein localises to the axoneme, consistent with localisation of its *Chlamydomonas* orthologue FAP161 as a microtubular inner protein of the A-tubule of outer doublet microtubules. In both analysed vertebrate species, the disruption of *Cfap161* did not result in phenotypes related to dysfunctional motile cilia. Comparison of mRNA from wild type and *Cfap161*-mutant mouse testis showed stabilisation or enhanced transcription of *Cfap161* mRNA in mutants and dysregulation of more than 300 genes including genes related to microtubules and cilia, microtubule motor activity and inner dyneins.

The absence of phenotypes related to dysfunctional motile cilia in both *Xenopus* and mouse came as a surprise given that morpholino-mediated knock down of *cfap161* (previously referred to as *C18H15orf26)* caused strong^37^ and mild^38^ ciliopathy phenotypes in zebrafish. Also, the interaction of CFAP161 with KIAA0556, a basal body and microtubule-associated protein which stabilises cytoplasmic microtubules in human cells and regulates ciliary A-tubule number in *C. elegans*, supported a ciliary function of CFAP161, although a truncation of the KIAA0556 protein was not associated with defective cilia structure or motility in ventricular ependymal cells in mice as well^41^. One potential explanation for the lack of *Cfap161* mutant phenotypes in *Xenopus* and mouse could be the presence of redundant genes. However, we did not find any evidence for the presence of *Cfap161* paralogs in both species. To investigate the function of *cfap161* in the frog *Xenopus*, we initially also used Morpholino oligomers (MOs) that interfere with either mRNA translation or splicing (TBMO or SBMO). However, only high MO doses resulted repeatedly in specimens with enlarged brain ventricles, oedema and compromised ciliation of epidermal MCCs, and we were unable to rescue any of these phenotypes with a tagged murine or untagged *Xenopus cfap161* mRNA (data not shown). Because these phenotypes were not observed after disruption of *cfap161* by CRISPR/Cas9 we attribute these phenotypes to unspecific effects of the used morpholinos at high concentrations. A ciliary function of *cfap161* in zebrafish that has been lost in *Xenopus* and mice cannot be ruled out at present, however it seems unlikely. Thus, it is possible that the discordant observations after morpholino knock down of *cfap161* in zebrafish^37,38^ reflect unspecific effects similar to those we observed in *Xenopus*.

Genetic compensation of mutations is increasingly observed in various species (reviewed in^45^). *Xenopus cfap161*-crispants showed downregulation of *cfap161* mRNA (Fig. S6Cb,b′) suggesting nonsense-mediated decay. mRNA degradation is known to trigger genetic compensatory mechanisms in zebrafish embryos and mouse cells^46^. Thus, in principle, the absence of a motile cilia related phenotype in *Xenopus* crispants might be the result of genetic compensation triggered by *cfap161* mRNA decay. However, the absence of a specific effect in *cfap161* morphants argues against this possibility. Also, our RNA-seq data from the mouse do not support the action of a compensatory mechanism triggered by nonsense-mediated mRNA decay because *Cfap161* mRNA was stabilised rather than destabilised in mutants. Thus, if genetic compensation underlies the absence of a phenotype in *Cfap161*-mutant mice the mechanism cannot depend on mRNA degradation^46^. mRNAs bearing a premature termination codon as is the case in our *Cfap161*^*∆ex2,3*^ allele can trigger genetic compensation by promoting transcription of homologous genes^47^. However, as stated before, no *Cfap161* paralogs were found in the mouse genome making this possibility unlikely.

The high number of statistically significantly dysregulated transcripts in *Cfap161*-mutant testis resulting from mutation of a gene without known transcriptional activity is surprising as is the abundance of these dysregulated genes on chromosome 7, where also *Cfap161* is located. Analysis of gene ontology and GO molecular function and GO cellular component datasets suggested a potential effect of CFAP161 loss of function on the expression of other genes including genes related to microtubules and cilia, microtubule motor activity and inner dyneins. A conclusive interpretation of the RNA-seq results seems difficult as to the precise mechanism of why expression of so many genes is affected and how that might affect the loss of *Cfap161* phenotype and potentially buffer the *Cfap161* mutation.

While protein complexes bound to the inner surface of doublet microtubules were identified in diverse species (reviewed in^48^) not much is known about the biological functions of these proteins. In *Tetrahymena* RIB72A and the conserved RIB72B are MIPs that bind to the inner surface of A-tubules. Deletion of either protein caused disruption of distinct MIP complexes, severe structural defects of the A-tubule and abnormal ciliary beating indicating MIP functions in stabilisation of doublet microtubules and possibly regulation of ciliary beating^49^. In contrast, single deletion of the conserved B-tubule MIPs FAP45 and FAP52 had a slight or no effect, respectively, on swimming velocity and ciliary beat frequency in *Chlamydomonas*, whereas only deletion of both MIPs significantly reduced both parameters and destabilised B-microtubules, which was suggested to reflect a “fail-safe” mechanism acting in the stabilisation of doublet microtubules^50^. Such a “fail-safe” mechanism could possibly also explain the apparent absence of a ciliary phenotype in *Cfap161* mutants. We cannot rule out that disruption of *Cfap161* causes subtle defects of stability or function of motile cilia. Such defects, if present, might sensitise cilia for other mutations or only manifest themselves under other conditions impinging on ciliary function.

## Material and methods

### Ethics approval

Mouse and *Xenopus laevis* handling and husbandry was in accordance with the German regulations (Tierschutzgesetz) and for mice approved by the ethics committee of Lower Saxony for care and use of laboratory animals (LAVES, Niedersächsisches Landesamt für Verbraucherschutz und Lebensmittelsicherheit), and for frogs by the Regional Government Stuttgart, Germany (A379/12 Zo ‘Molekulare Embryologie’). Mice were kept in the central animal facility of Hannover Medical School (ZTL) as approved by the responsible Veterinary Officer of the City of Hannover. All experiments were performed in accordance with the relevant guidelines and regulations and in compliance with the ARRIVE guidelines.

### Statistical analyses

Statistical analyses were done using Prism7 for Student’s paired t-test. The chi-square or Wilcoxon-Match-Pair test was done in RStudio (The R project for statistical computing; http://www.r-project.org/). The used tests are indicated in the respective figure legends. Statistical analysis for proteomics data was done using Perseus.

### Mouse methods

#### Experimental animals

*Foxj1-*mutant *(Foxj1*^*lacZ*^)^22^, Zp3:Cre^42^, and FLPe mice^51^ were described previously. *Cfap161*^*loxP*^ mice were generated by Cyagen (Cyagen Biosciences, California, USA). The neo cassette was flanked by FRT sites and removed by FLP-mediated recombination. Exon 2 and 3 were deleted in the germline of *Cfap161*^*loxP*^; Zp3:Cre^42^ double heterozygous females. *Cfap161*^*∆ex2,3*^ mice were analysed on a hybrid (CD-1//129/Sv) genetic background.

#### Mouse genotyping

*Cfap161*-mutant and wild type mice were genotyped by PCR with primer pairs:

- Cfap161-loxP-F1: 5′-ACCTTTGCTACCCGAGGTAGTTC-3′, Cfap161-loxP-R1: 5′-ATGCATAAGGAAGGGAAGGATAGG-3′, 312 bp wild type, and 372 bp *Cfap161*^*loxP*^ product.
- Cfap161-loxP-F1: 5′-ACCTTTGCTACCCGAGGTAGT-3′, Cfap161Neo del-R1: 5′-CCTGCGGATCATTTCCAAAACCTC-3′, 515 bp *Cfap161*^*∆ex2,3*^ product.

#### Collection, embedding, and sectioning of mouse tissues

Mice were killed by cervical dislocation, tissues dissected, fixed overnight at 4 °C in 4% PFA, 100% methanol or 100% methanol/DMSO (4:1), if necessary decalcified in 0.5 M EDTA for 2 weeks exchanging EDTA every other day, dehydrated, embedded in paraffin according to standard procedures and sectioned at 5 or 10 µm. For standard histology the lungs were not inflated, but wt and mutant were equally treated. Mice used for transmission electron microscopy (TEM) were perfused with fixative followed by dissection of the organs and embedded and analysed as described^52^. See “Supplemental Material and Methods” for details.

#### Section in situ hybridisations (SISH)

SISH were performed on 10 µm sections of PFA fixed and paraffin-embedded tissues as described in^53^. DIG-labelled RNA probe was produced from FANTOM plasmid ZX00121F10^54^ using the Roche DIG RNA labelling system.

#### Whole mount in situ hybridisation (WISH)

WISH were performed on E7.5–8.0 old mouse embryos as described^29^.

#### Histological staining

5 µm sections of PFA fixed and paraffin-embedded tissues were stained with haematoxylin and eosin (HE) according to standard procedures, or with PAS using the Periodic Acid-Schiff Kit (Sigma Aldrich) according to manufacturer’s instructions.

#### Cell culture

Murine tracheal epithelial cells (mTECs) were isolated and cultured at air liquid interface (ALI) as described^55^, and cultured for up to 10 days. CHO cells were cultured in DMEM/F12 containing 10% FCS, Pen/Strep, and 2 mM Glutamax (Gibco). CHO cells were transfected using Perfectin (Genlantis) according to manufacturer’s instructions.

#### RNA isolation and RT-PCR

Total RNA was isolated using Direct-zol RNA MiniPrep Kit (ZYMO) according to the manufacturer’s instructions. cDNA was produced using the Superscript-II Reverse Transcriptase kit (Thermo Fisher Scientific). PCRs were performed using primer combinations: mouse *Hprt* (exon 7–9; 249 bp) (5′-CACAGGACTAGAACACCTGC-3′; 5′-GCTGGTGAAAAGGACCTCT-3′), mouse *Cfap161* (exon 4–6; 230 bp) (5′-ATAGCAGCAGGACTGGAAGGCAAG-3′; 5′-GCTCGGTTTGTGTGACGATGATAG-3′), mouse *Foxj1* (exon 2–3; 432 bp) (5′-CTTCTGCTACTTCCGCCATGC-3′; 5′-TCCTCCTGGGTCAGCAGTAAGG-3′). *Hprt*-RT-PCR was used to validate the integrity of the RNA.

#### RNA isolation and RNA-seq

Total RNA was isolated from single testis without epididymis from 3 different 11–17 weeks old wild type and 3 different 11–13 weeks *Cfap161*^*∆ex2,3/∆ex2,3*^ males using the Direct-zol RNA MiniPrep Kit (ZYMO) according to manufacturer’s instructions. RNA integrity was first validated by gel electrophoresis, then further analysed by Eukaryote Total RNA Nano and Bioanalyser. Details concerning library generation, quality control and quantification as well as details for the sequencing run and raw data processing can be found in the “Supplementary Materials and Methods”.

#### Generation of antibodies

Monoclonal antibodies (MAbs) against mouse CFAP161 were generated as described in^33^ by immunisation of rats with the peptide RMGNWNEDVYLEEERM (pI; aa11-26). MAbs that reacted specifically with CFAP161 were further analysed by Western blots and indirect immunofluorescence. α-CFAP161 pI clone 8F9 was further characterised (see Fig. S3 and “Supplemental Methods” for details) and used in this study. Rabbit polyclonal antibodies against mouse CFAP161 epitope ELLIQRNRRVKKNIL (pII; aa37-51) and YLDSHKVEKPKNQW (pIII; aa254-267) in two different animals (a1 and a2) were generated and affinity purified by BioGenes (BioGenes GmbH, Berlin).

#### Immunofluorescence staining

Sections (5 µm) were deparaffinised, rehydrated and antigens unmasked by boiling for 20 min in 10 mM Tris–HCl pH 9.5, 1 mM EDTA. Unspecific binding was blocked by 5% FCS in PBS. Primary antibodies were incubated at 4 °C overnight, secondary antibodies for 1 h at room temperature. Antibodies were diluted in blocking solution: α-acetylated α-tubulin (ac-TUB) (Sigma Aldrich T6793) 1:1000; α-CFAP161 pI 1:50; α-CFAP161 pII 1:2000; α-mouse-Alexa633 (Invitrogen A21052) 1:500; α-mouse-Alexa555 (Life Technologies A31570) 1:500; α-mouse-Alexa555 (Invitrogen A21424) 1:500; α-rat-Alexa488 (Invitrogen A21208) 1:500; α-rabbit-Alexa488 (Invitrogen A11034) 1:500; and α-rabbit-Alexa555 (Invitrogen A21429) 1:500. DAPI (1 µg/ml, Applichem) and Lectin-PNA-Alexa488 (Thermo Fisher L-21409) 1:500 were incubated together with secondary antibodies.

#### Western blot analysis

SDS-PAGE and Western blotting were carried out according to standard procedures. The following amounts of total proteins were loaded per lane: CHO cells (Fig. 2B and Fig. S9A) and testis or epididymis lysate (Figs. 2B, 5B, Fig. S9A,C) 15–20 µg. For mTEC ALI cultures (Fig. 2C and Fig. S9B) 1/10 of the lysate of a confluent 24-well transwell insert was loaded, which amounts to 2.5–4 µg according to^55^. Blots were blocked with 5% milk in PBS/0.1% Tween20. Antibodies were diluted in blocking solution: α-β-TUBULIN (β-TUB; Sigma; T7816) 1:250,000, α-IFT88 (Proteintech; 13967-1-AP) 1:1000, α-CFAP161-pI 1:100, α-CFAP161-pII 1:2000, α-mouse-POD (Amersham NA931) 1:10,000; α-rabbit-POD (Amersham NA934) 1:10,000, and α-rat-POD (Amersham NA935) 1:10,000. Western blots were developed using WesternBright Quantum (Advansta).

#### Yeast two-hybrid (Y2H) assay to validate CFAP161-KIAA0556 interaction

Yeast two-hybrid assays were performed according to the manufacturer’s protocol (Matchmaker Gold Yeast Two-Hybrid System 630489, Clontech). For details see “Supplemental Methods”.

#### Immunoprecipitations (IPs) and mass spectrometry (MS)

CFAP161 was immunoprecipitated from wild type mouse testis with epididymis using the Thermo Scientific Pierce crosslink IP kit using α-CFAP161 pII or with rabbit-IgG as controls. In each experiment (n = 3) the total lysate of one homogenised (160–200 mg) testis and epididymis (~ 8–10 mg total protein) was incubated either with IgG or affinity-purified α-CFAP161 pII crosslinked to resin. 1 mg of protein was loaded to 40 µl settled resin as suggested by the manufacturer’s instructions (in practice, 2 mg of total protein was loaded per column containing 80 µl of resin) in n = 6 IgG control IPs and n = 7 CFAP161 IPs. Immunoprecipitated CFAP161 complexes were isolated, purified, and subsequently analysed by LC–MS/MS as described^34^.

### *Xenopus* methods

#### Whole mount in situ hybridisation (WISH)

WISH were performed on MEMFA fixed embryos covering the first week of development. DIG-labelled RNA probe was produced from I.M.A.G.E. clone plasmid IRBHp990H1063D^54^ using the Roche DIG RNA labelling system.

#### Generation and verification of crispants

*cfap161* sgRNAs targeting exon 1 and exon 3 were designed using CRISPRscan. DNA templates were created using the Promega Pfu proofreading polymerase for oligo extension reaction. sgRNA synthesis utilised the Invitrogen mMESSAGE mMACHINE T7 Transcription Kit in combination with the Invitrogen MEGAclear Transcription Clean-Up Kit. 300 ng *foxj1*^33^ or 150 ng of each *cfap161* sgRNAs were preassembled with 1 ng PNABio Cas9 protein from *Streptococcus pyogenes* with NL and injected at the 1 cell stage. Genome editing was confirmed via Synthego ICE analysis (https://ice.synthego.com) after direct sequencing of PCR products from pooled stage 45 embryos using the following primer combinations: *cfap161* target site exon 1 (5′-CGTCGCCTTGGCAACTGATA-3′; 5′-TGGGGGTCGGATTTTCAATG-3′), *cfap161* target site exon 3 (5′-TTTGGGGATGTGGTGATGCT-3′; 5′-CGTCACACTCAGTGACATCAT-3′).

#### CFAP161/Cfap161 localisation

20 pg of either pCS2 + EmGFP-*Cfap161,* pCS2 + HA-*cfap161 or* pCS2 + *cfap161*-HA were co-injected with 20 pg pCS2 + *cetn4*-RFP or pCS2 + GFP-*cetn4* at the 4 cell stage into the ventral marginal zone to target the epidermis. Embryos were fixed in MEMFA and stained with Thermo Fisher Scientific Alexa Fluor 405 Phalloidin to mark the F-actin of the cell borders before fluorescence imaging. CFAP161- and CETN4-localisation was shown by direct fluorescence.

#### High-speed video microscopy of larval epidermal cilia

Videos were recorded at stage 32 embryos using a Zeiss Axioskop 2 mot plus microscope. Embryos were mounted on a slide containing a chamber constructed from duct tape. The most ventral part of the belly was recorded with a high-speed Hamamatsu video camera Orca flash 4.0 at 800 frames per second (fps) for 1 s to analyse ciliary beating. Ciliary flow was analysed using 1 μm fluorescent beads (Invitrogen FluoSpheres; 1:2000). Beads were added to the culture medium (0.1 × MBSH) and specimens were imaged using a Zeiss Axiocam HSm camera at 175 fps. Evaluation of CBF and bead transport was previously reported in^33^.

#### Documentation

WISH, SISH, histological stainings and IHC on sections of all mouse experiments were documented with a Leica DM5000 microscope equipped with a Leica DFC300FX digital camera and the used software was Leica FireCam (Version 1.9.1). Immunofluorescence staining of mouse tissue sections were photographed with the Leica DMI6000 microscope equipped with a Leica DFC350FXR2 camera and the Leica Application Suite X software. Images were processed in Adobe Photoshop CS4 or CC, or FIJI ImageJ version 2.0.0, figures assembled using Adobe Illustrator CS4, CS6 or CC. *Xenopus* WISH was imaged with a Zeiss SteREO Discovery.V12 or if sections were made with a Zeiss Axioskop 2 mot plus each equipped with an AxioCam HRc in combination with AxioVision 4.7. Fluorescence imaging of *Xenopus* was performed on a Zeiss AxioObserver with a LSM700 and Zeiss ZEN black software. CBF was documented with a Zeiss Axioskop 2 mot plus equipped with a high-speed Hamamatsu video camera Orca flash 4.0 and Zeiss ZEN blue. CGF analysis was done with a Zeiss Axioskop 2 mot plus combined with a Zeiss AxioCam HSm camera and Zeiss AxioVision 4.7. CBF and CGF was assessed using the ImageJ plugin Particle Tracker^56^. All trajectories were further analysed using a custom-made program written in RStudio which calculated the velocity of each particle track^57^.

## Supplementary Information

Supplementary Information 1.

Supplementary Information 2.

Supplementary Information 3.

## Data Availability

The data generated or analysed during this study are included in this article (and its Supplementary Information files). The full mass spectrometry proteomics data have been deposited to the ProteomeXchange Consortium via the PRIDE^58^ partner repository with the dataset identifier PXD022420.
