# Supplementary material for: The highly conserved FOXJ1 target CFAP161 is dispensable for motile ciliary function in mouse and Xenopus
Source: Sci Rep. 2021 Jun 25;11:13333. doi: 10.1038/s41598-021-92495-3 (PMC8233316; doi:10.1038/s41598-021-92495-3)
Supplement: Supplementary file 1 — Supplementary Information 1. [file 41598_2021_92495_MOESM1_ESM.pdf]

SUPPLEMENTARY INFORMATION

The highly conserved FOXJ1 target CFAP161 is dispensable for motile ciliary function in mouse and *Xenopus*

Anja Beckers, Franziska Fuhl, Tim Ott, Karsten Boldt, Magdalena Maria Brislinger, Peter Walentek, Karin Schuster-Gossler, Jan Hegermann, Leonie Alten, Elisabeth Kremmer, Adina Przykopanski, Katrin Serth, Marius Ueffing, Martin Blum and Achim Gossler

This file contains Supplementary Figures S1-S9, Supplementary Tables S1, S3-5, S7, S8 and Supplementary Material and Methodes.

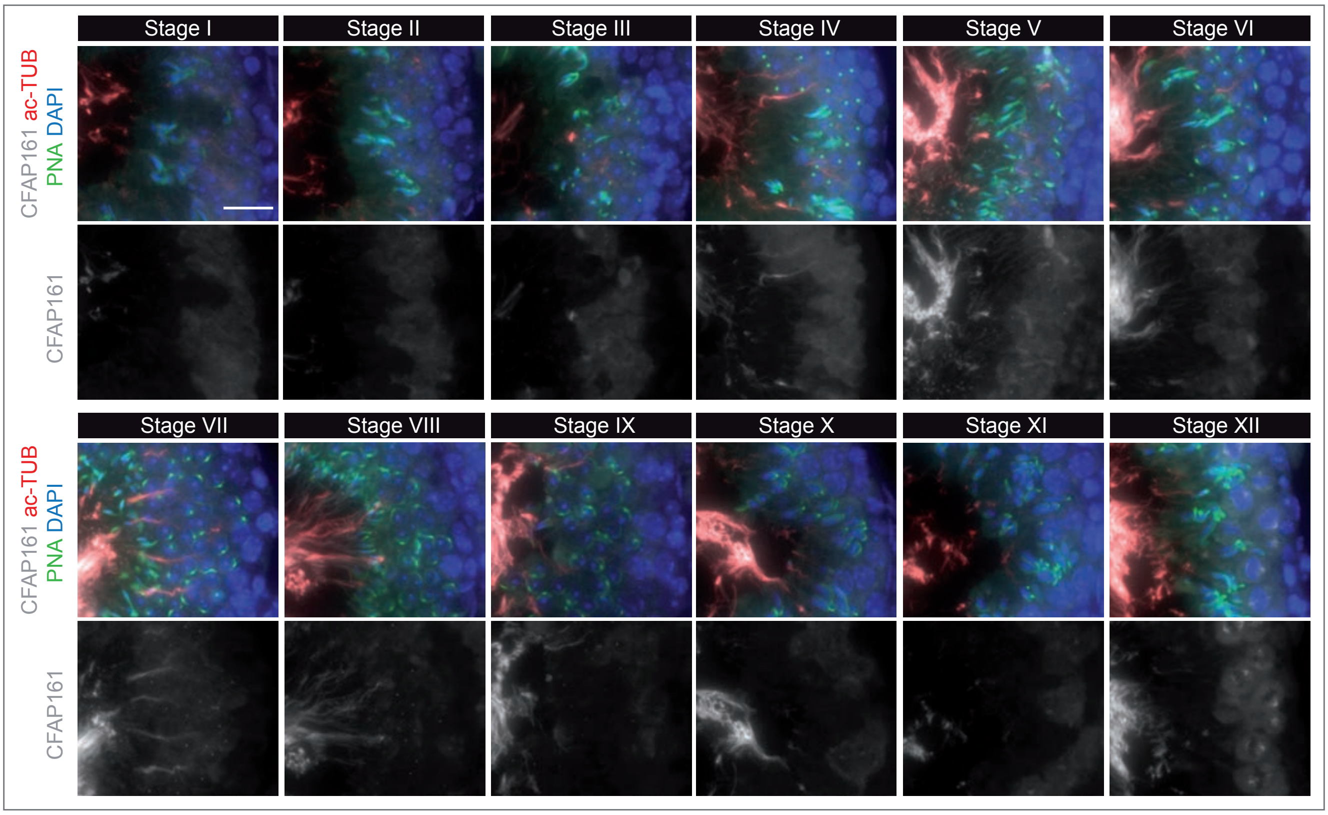

Fig. S1. CFAP161 distribution in mouse spermatids. Sections of murine wild type testis stained for acrosomes (PNA; green), nuclei (DAPI; blue), axonemes (α-ac-TUB; red) and CFAP161 (α-pII antibody; white). Upper pictures show the merge of all channels and the lower pictures the single CFAP161 channel. Stage I-XII on the top indicate the stages of the epithelial cycle. Lumen of the sectioned seminiferous tubules is oriented to the left.
Scale bar = 25 µm.

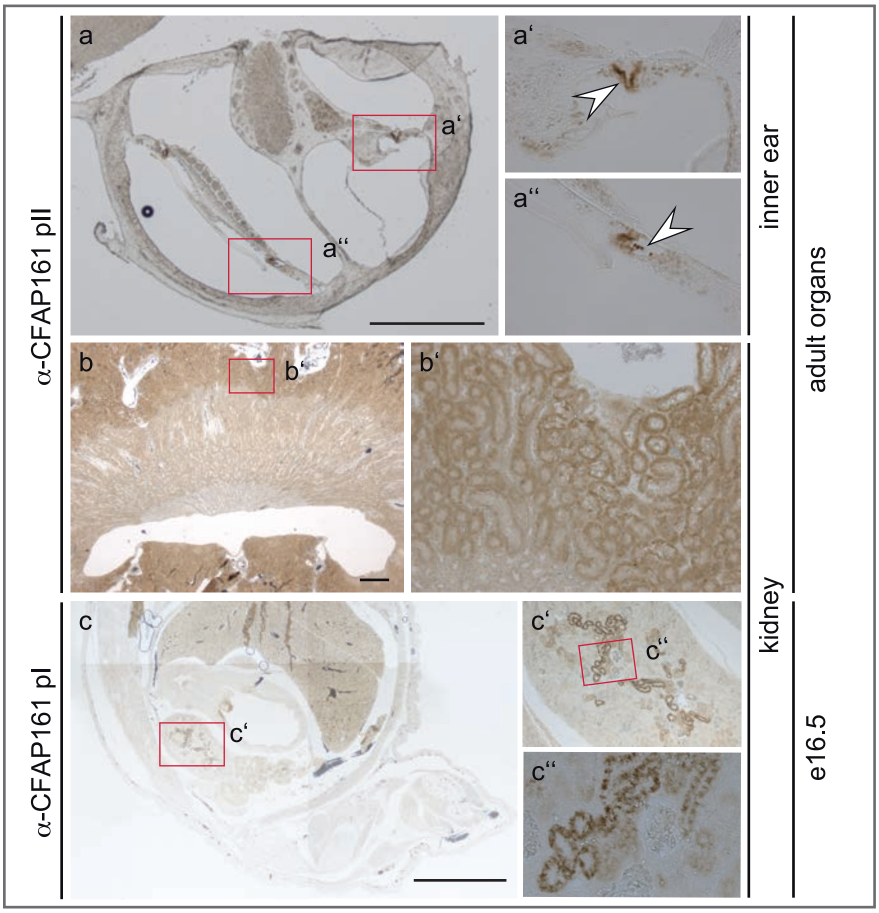

Fig. S2. CFAP161 in mouse tissues with immotile cilia. CFAP161 expression was detected in adult inner ear (a), representing mainly the inner and outer hair cells (white arrowheads in a' and a'') and in kidney cells (b) mainly localised to the collecting duct system. CFAP161 is visualised by indirect DAB-staining and polyclonal α-pII antibody. In sections of mouse embyos E16.5 (c) strong CFAP161 staining is observed in the developing collecting duct system (c), harbouring immotile (primary) cilia. CFAP161 is visualised by indirect DAB-staining and polyclonal α-pI antibody. Red boxed areas in (a,b,c,c') indicate the regions shown at higher magnification in (a',a'',b',c',c''). Scale bars: Aa,c = 1 mm; Ab = 100 µm.

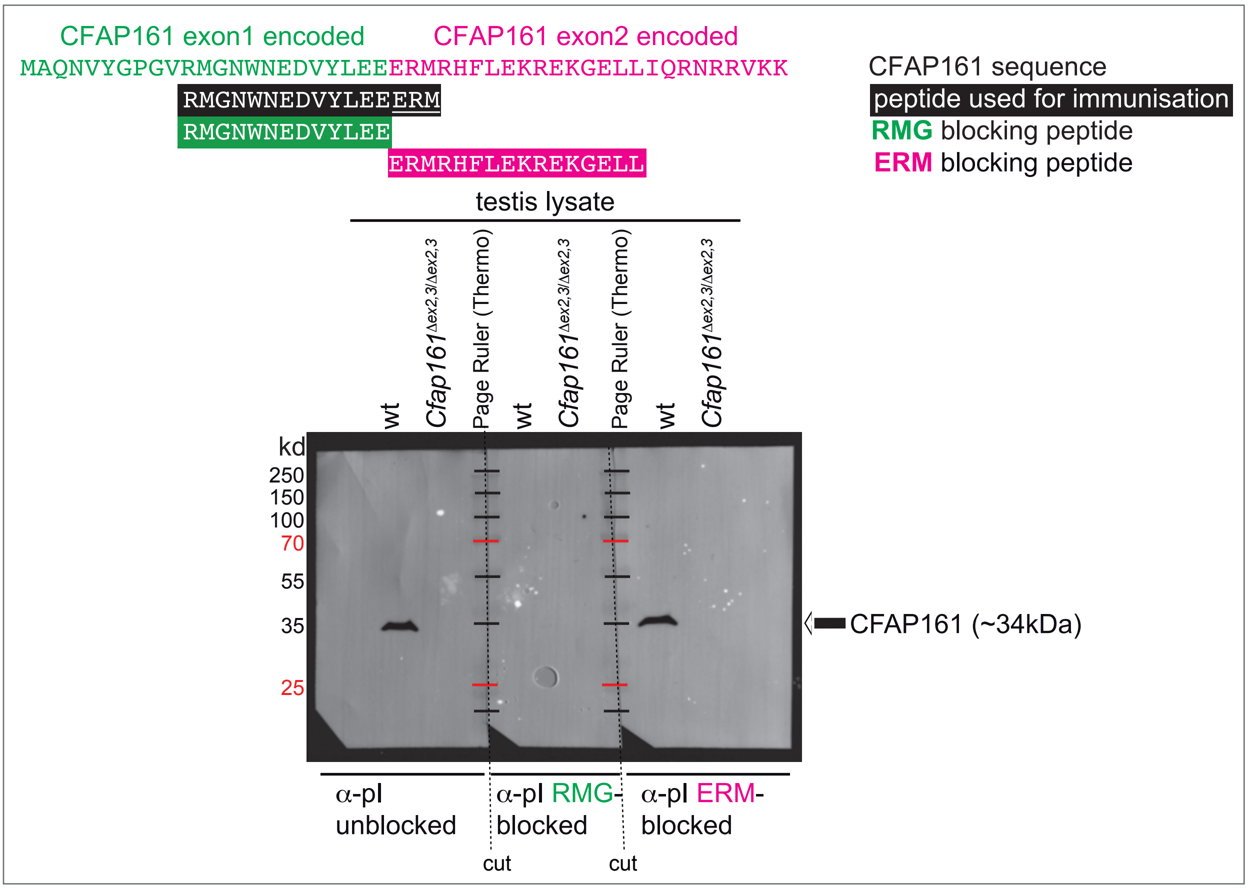

Fig. S3. Blocking experiment showed that the α-pI recognition site is encoded by exon1 of CFAP161. The monoclonal rat antibody (α-pI; clone 8F9) was generated by immunisation with the peptide RMGNWNEDVYLEEERM (black box with white letters) that is mainly encoded by exon1 (green coloured sequence) except for the last three amino acids of the peptide (underlined) that are encoded by exon2 (magenta coloured sequence). To identify the epitope recognised by α-pI two blocking peptides were used (RMG, green box; ERM, magenta box). Only blocking with RMG interfered with the recognition by α-pI, whereas, blocking ERM did not prevent binding of α-pI to immuno-blotted CFAP161 resembling the unblocked conditions.

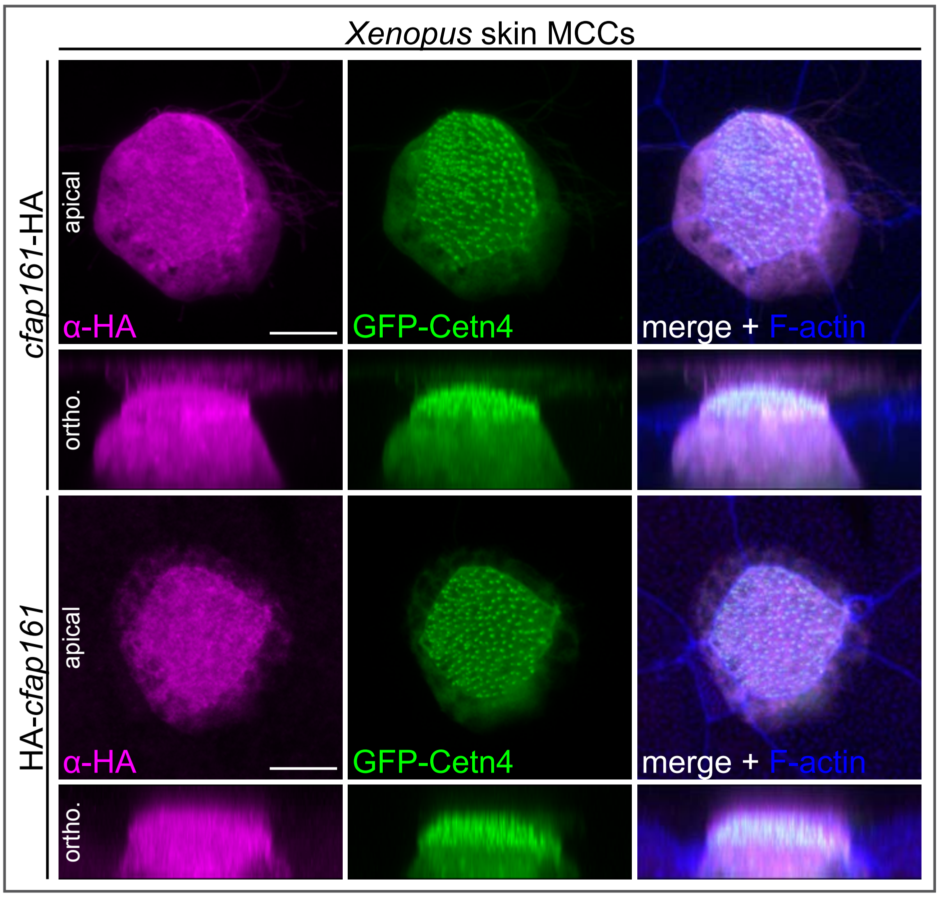

Fig. S4. Subcellular localisation of *Xenopus* Cfap161

N- or C-terminally HA-tagged Cfap161 was expressed in *Xenopus* skin MCCs in combination with GFP-Cetn4 to label basal bodies. Cfap161 signals were found throughout the cells but did not specifically accumulate in cilia. Apical and orthogonal views as indicated. Scales bars = 100 µm.

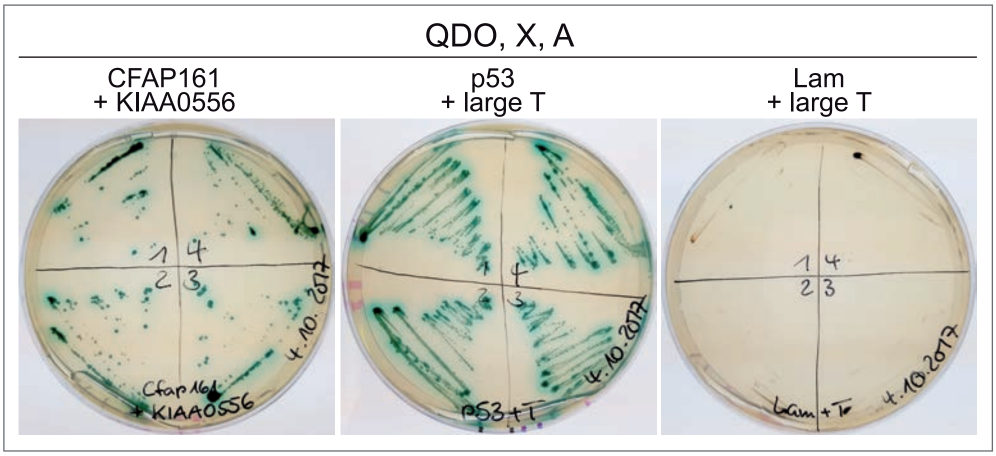

Fig. S5. Yeast Two-Hybrid assay demonstrate direct interaction of CFAP161 and KIAA0556. After mating the haploid yeast strains Y2HGold containing the Gal4 BD bait construct (CFAP161) and Y187 containing Gal4 AD prey construct (KIAA0556) cells were selected on DDO (SD/-Trp/-Leu) medium for diploid colonies. To confirm interaction between bait and prey at least four different colonies from DDO were picked on quadruple dropout medium (QDO, SD/-Trp/-Leu -Ade/-His) supplemented with X-α-Gal (X) and Aureobasidin A (A). Mating with p53 and large T was used as a positive control and Lam with large T as a negative control in the assay.

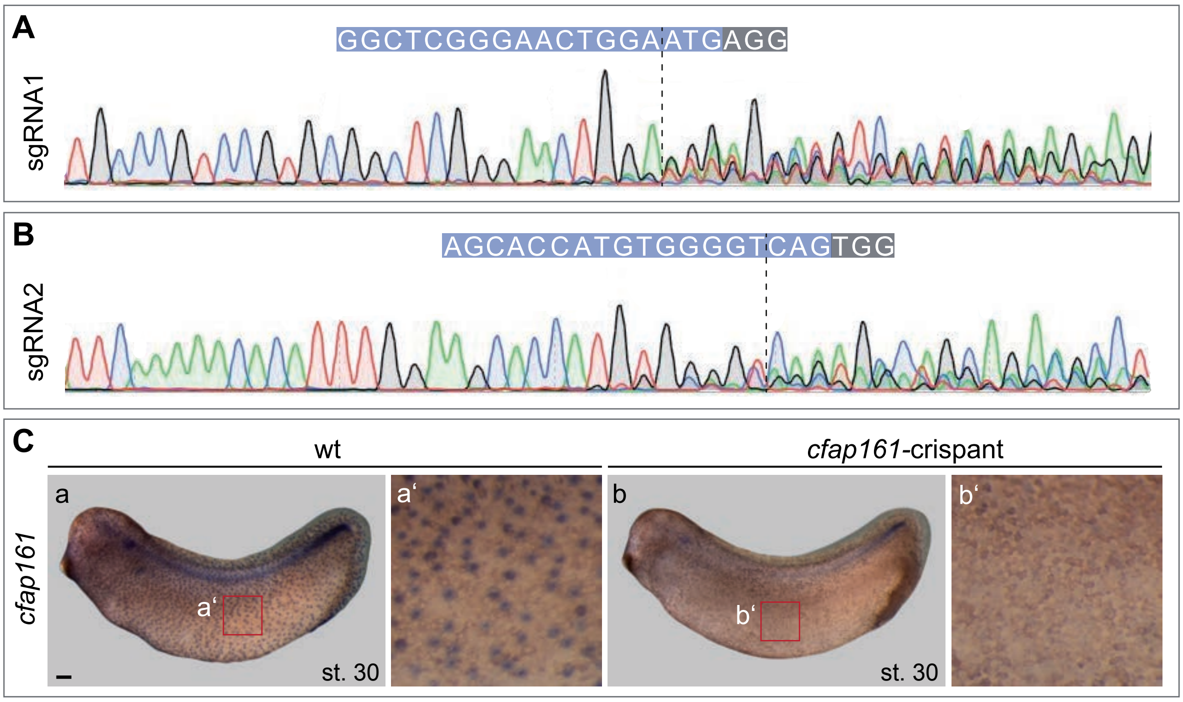

Fig. S6. Genome editing of the *cfap161* locus in *Xenopus*. (A) Sequence of the *cfap161* L-allele of genome edited specimens injected with sgRNA1, which targets exon 1. **(B)** Sequence of the *cfap161* L-allele of genome edited specimens injected with sgRNA2, which targets exon 3. **(C)** *cfap161* transcripts were reduced in *cfap161*-crispants (b) at stage 30, compared to wt (a) with a focus on the skin. Red boxed areas in (a,b) indicate the regions shown at higher magnification in (a‘, b‘). Scale bar: C = 150 µm.

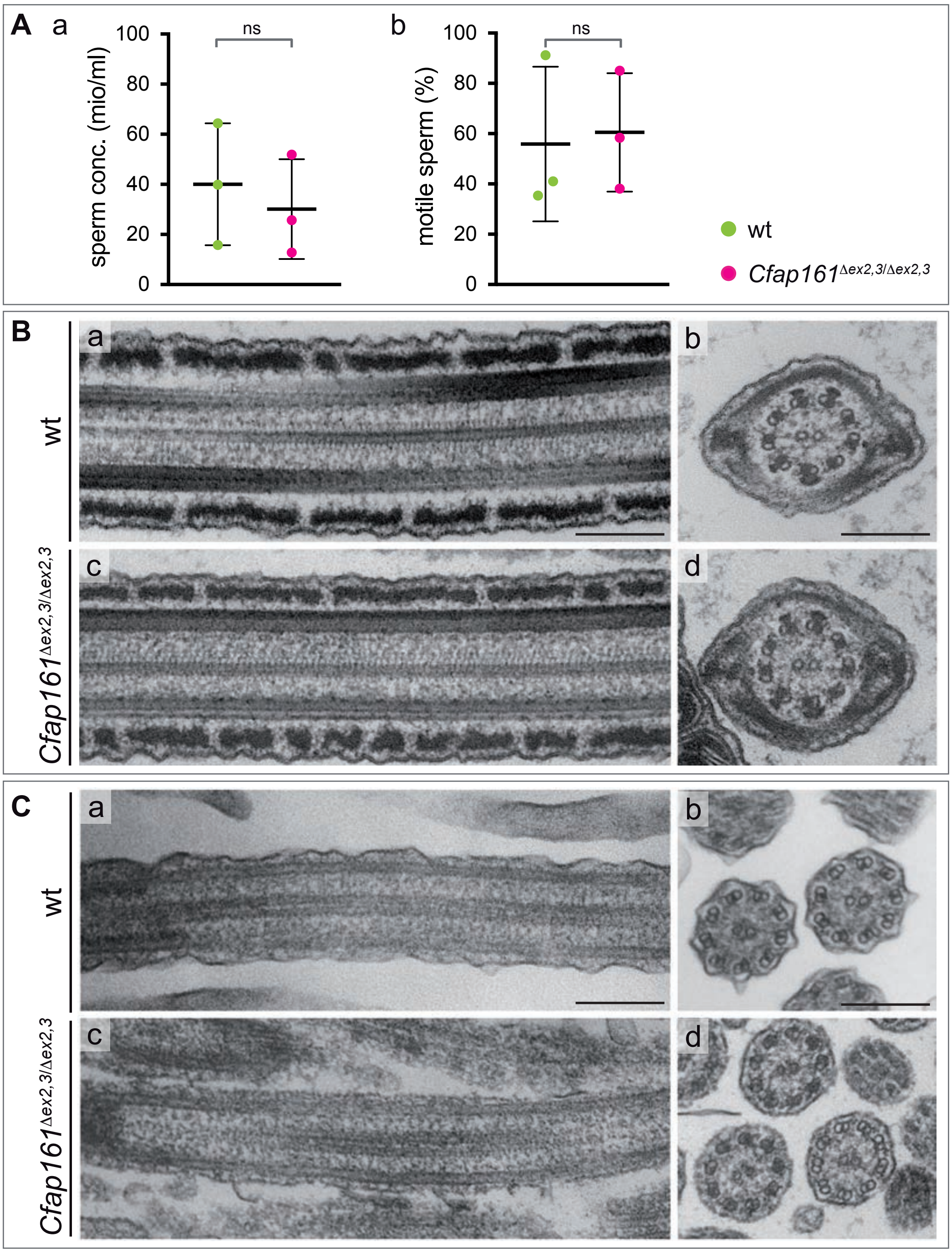

Fig. S7. Analysis of wild type and *Cfap161*$^{\Delta ex2,3/\Delta ex2,3}$ sperm flagella and lung cilia. (A) Computer assisted sperm analysis (CASA) shows no significant change in sperm concentration (a) and sperm motility (b) in *Cfap161*$^{\Delta ex2,3/\Delta ex2,3}$ mutants compared to wild type. Each dot represents the average of 8 or 12 measurements of one specimen analysed (wt n=3; *Cfap161*$^{\Delta ex2,3}$ n=3). Raw data of all measurements are shown in Table S5. Graphs display respective values with mean and s.d.; Student's paired t-test. **(B)** Electron microscopy of wild type (a,b) and *Cfap161*$^{\Delta ex2,3/\Delta ex2,3}$ mutant (c,d) longitudinally sectioned (a,c) and cross-sectioned (b,d) spermflagella, displaying axonemal profiles of wild type and mutant principal pieces with normal organised microtubules surrounded by fibrous sheaths.**(C)** Electron microscopy of wild type (a,b) and *Cfap161*$^{\Delta ex2,3/\Delta ex2,3}$ mutant (c,d) longitudinally sectioned (a,c) and cross-sectioned (b,d) cilia in lung airways, displaying axonemal profiles of wild type and mutant cilia with normal organised microtubules.
Scale bars: B,C = 200 nm.

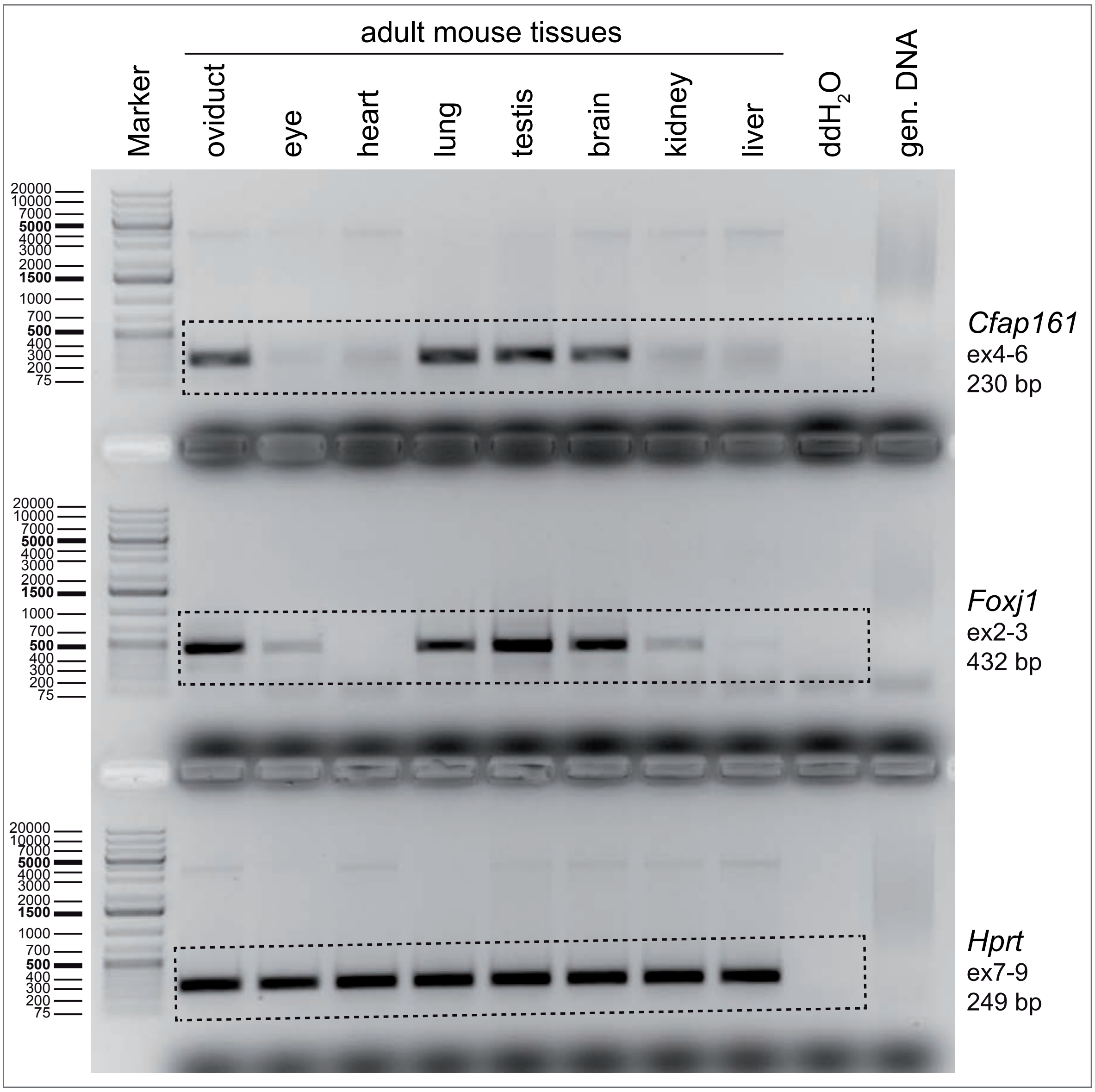

Fig. S8. Complete gel of RT-PCR shown in Fig. 1A. PCRs on RNA isolated from various wild type adult mouse tissues as indicated at the top. Stippeled boxes indicate the gel areas shown in Fig. 1A. Product sizes: *Cfap161* exon4-6, 230 bp; *Foxj1* exon2-3, 432 bp; *Hprt* exon7-9, 249 bp. GeneRuler 1kb Plus (Thermo) was used as standard.

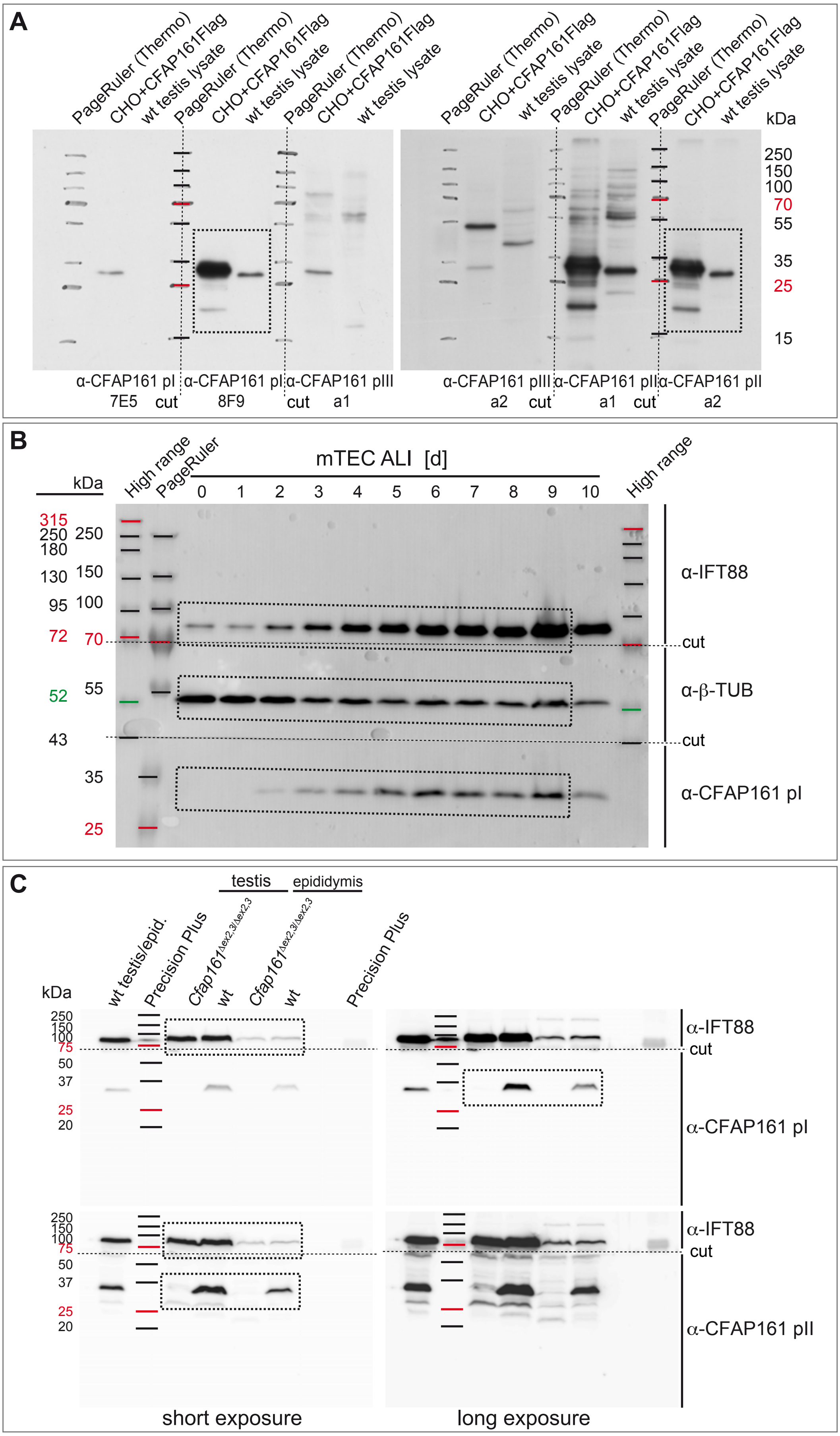

Fig. S9. Full-size Western blots of CFAP161-expressing CHO cells, mTEC lysates as well as mouse testis and epididymis lysates.
(A) RAW data to Fig. 2B. Each blot was cut into three pieces which were incubated with different α-CFAP161 antibodies. α-CFAP161 pI 8F9 and α-pII a2 gave best signals and were used for further experiments. 7E5 is an additional MAb against pI. α-CFAP161 pIII are PAbs against another peptide. **(B)** RAW data to Fig. 2C. **(C)** RAW data to Fig. 5B. The cropped areas used for the main figures are marked by stippeled boxes.

Table S1. Similarity matrix of CFAP161 amino acid sequences from different species.
Sequences were aligned using ClustalW (v1.83; multiple sequence alignment; Pairwise Alignment Mode: Slow; Pairwise Alignment Parameters: Open Gap Penalty = 10.0, Extend Gap Penalty = 0.1, Similarity Matrix: gonnet; Multiple Alignment Parameters: Open Gap Penalty = 10.0, Extend Gap Penalty = 0.2, Delay Divergent = 30%, Gap Distance = 4).

		** Identity Scores (%) **							
**CFAP161**		*H. sapiens*	*M. musculus*	*C. japonica*	*X. tropicalis*	*D. rerio*	*B. lanceolatum*	*C. intestinalis*	*C. reinhardtii*
H. sapiens NP_775799	** Similarity Scores (%) **	100.0	72.4	48.0	47.2	39.7	38.6	42.1	18.5
M. musculus NP_083611		85.5	100.0	47.9	45.4	36.3	35.0	40.1	16.9
C. japonica XP_015728586		67.2	64.7	100.0	47.3	38.6	37.8	43.7	16.5
X. tropicalis *NP_001120035*		64.4	62.8	64.1	100.0	45.7	41.9	49.4	17.5
D. rerio NP_001017774		60.3	59.2	58.1	62.9	100.0	38.3	41.6	18.7
B. lanceolatum XP_019614576		54.5	53.0	52.2	57.2	55.3	100.0	50.6	19.9
C. intestinalis XP_002124415		61.4	57.4	58.6	67.5	61.6	66.5	100.0	20.0
C. reinhardtii EDP08927		31.6	30.8	29.4	30.1	31.4	35.4	32.6	100.0

Table S3: Raw data of CBF on *Xenopus* epidermal MCCs shown in Fig. 4Ea.

Ciliary beat frequency (CBF in Hz) of skin cilia determined for 15 control and 15 crispant individuals of 3 independent experiments. Statistical analysis was done via Wilcoxon-Match-Pair test in RStudio.

	co														
animal	1	2	3	4	5	6	7	8	9	10	11	12	13	14	15
CBF	19.4	20.8	23.8	22.4	22.6	21.8	21.8	22	20	22.5	22.8	23.6	19.4	26.4	23.2
	20.6	20.6	18.8	23.8	22.4	22.4	22	20.8	21.2	21.4	20.2	23.4	20	22.6	22.2
	20.4	21.8	20.2	24.8	20.2	26	21.8	18.4	21.6	21.6	21.2	24	17.8	23.4	23.2
	19.6	22.6	21.2	24.2	22.2	23.8	22	19.2	21.6	19.8	23.2	21.8	20.8	17.4	24
	20.6	22.6	20.8	20.8	24.2	27.2	17.6	21	22.6	21.8	23	22	21.8	18.4	24.4
Mean	20.12	21.68	20.96	23.2	22.32	24.24	21.04	20.28	21.4	21.42	22.08	22.96	19.96	21.64	23.4
SD	0.576194	0.954987	1.829754	1.606238	1.425482	2.312574	1.925617	1.453272	0.938083	0.995992	1.316055	0.993982	1.505988	3.71322	0.848528

	sgRNA1+2														
animal	1	2	3	4	5	6	7	8	9	10	11	12	13	14	15
CBF	24.2	19.6	19.4	21	21.8	21.8	24.4	21	20.6	22.8	23.6	23.4	21.8	20.4	21.8
	24.8	41.6	22	14.4	22.8	21	22	20.6	21.8	21.2	22.8	25.2	20.4	20.2	21.8
	21.8	21.4	19.8	21.6	21	22.6	22.4	23.6	22.6	22	22.2	24.6	23.2	19.8	19.8
	19.8	20.4	23.4	22.2	20.2	21.8	23.8	24.6	22.2	21.2	22.2	28	20.6	21.6	16.2
	21.6	18.6	21	19.2	21.4	23.4	22.8	23.6	22	20.4	24	25.4	20	17.8	20
Mean	22.44	24.32	21.12	19.68	21.44	22.12	23.08	22.68	21.84	21.52	22.96	25.32	21.2	19.96	19.92
SD	2.04646	9.714525	1.634625	3.157847	0.963328	0.91214	0.995992	1.769746	0.753658	0.91214	0.817313	1.688787	1.30384	1.381304	2.287357

Table S4. Raw data of CGF on *Xenopus* epidermal MCCs shown in Fig. 4Fa.
Mean velocity (μm/s) of cilia generated flow (CGF) analysed via bead tracking of 24 control and 24 crispant individuals in 3 independent experiments.

	wt	sgRNA 1+2
cilia generated flow (velocity in μm/s)	456.26	450.98
	481.14	264.57
	270.28	301.77
	469.07	370.54
	355.37	523.83
	391.57	485.09
	336.09	436.83
	453.04	445.98
	299	369.78
	359.81	246.1
	459.37	283.33
	397.65	453.16
	308.82	342.36
	162.11	266.29
	430.61	466.33
	337.29	373.86
	380.47	398.6
	448.2	468.29
	384.68	290.95
	449.45	444.23
	334.09	301.79
	288.41	366.46
	305.82	456.29
	519.49	497.52
mean	378.25	387.71
s.d.	83.25	84.22

Table S5. Computer-assisted sperm analysis (CASA) of wild type and *Cfap161*$^{\Delta ex2,3/\Delta ex2,3}$ sperm shown in Fig. S7A.

	animal ID	genotype	age (months)	sample_measurement	sperm conc. (mio/ml)	progressiveness (%)	motile sperm (%)	immotile sperm (%)	velocity (µm/s)
	1	wt	3	epididymis_M1	67.6	90.4	93	7	18
				epididymis_M2	57.6	82.9	85	15	17
				epididymis_M3	72.8	92.4	93	7	18
				epididymis_M4	61.2	89.9	91	9	18
				epididymis_M5	60.4	92	92.9	7.1	19
				epididymis_M6	61.2	90.6	92	8	20
				epididymis_M7	66	91.6	92.9	7.1	16
				epididymis_M8	68.4	88.6	90	10	17
**mean**					**64.4**	**89.8**	**91.2**	**8.8**	**17.9**
	2	wt	4	epididymis_M1	15.4	39.1	47	53	16
				epididymis_M2	17.2	27	31	69	16
				epididymis_M3	20	30.6	34.9	65.1	18
				epididymis_M4	17.4	31.9	35	65	21
				epididymis_M5	14.6	31.1	41	59	18
				epididymis_M6	13.8	24.8	34.1	65.9	18
				epididymis_M7	13.2	19.2	27	73	18
				epididymis_M8	14.6	26	33	67	19
**mean**					**15.8**	**28.7**	**35.4**	**64.6**	**18.0**
	3	wt	3	epididymis_M1	26.4	25.2	35	65	13
				epididymis_M2	27.2	29	36	64	14
				epididymis_M3	72.6	66	71	29	15
				epididymis_M4	81.2	65.7	71	29	16
				epididymis_M5	23	29.9	39	61	12
				epididymis_M6	36.6	6.7	14.1	85.9	13
				epididymis_M7	26.2	50.7	54	46	10
				epididymis_M8	25.8	5.5	8	92	11
**mean**					**39.9**	**34.8**	**41.0**	**59.0**	**13.0**
	4	*Cfap161*$^{\Delta ex2,3/\Delta ex2,3}$	3	epididymis_M1	44.6	59.7	62	38	18
				epididymis_M2	43.8	62.3	66.9	33.1	16
				epididymis_M3	50.2	82.2	85	15	21
				epididymis_M4	48.8	85.4	87	13	19
				epididymis_M5	66.8	97	97	3	19
				epididymis_M6	59.2	91.8	92	8	19
				epididymis_M7	43.8	86.8	89	11	21
				epididymis_M8	41	79.9	82.1	17.9	22
				epididymis_M9	54	88.2	90	10	21
				epididymis_M10	52.2	86.8	89	11	21
				epididymis_M11	64.6	94.8	96	4	21
				epididymis_M12	53.6	80.8	84	16	21
**mean**					**51.9**	**83.0**	**85.0**	**15.0**	**19.9**
	5	*Cfap161*$^{\Delta ex2,3/\Delta ex2,3}$	3	epididymis_M1	10.6	11.6	21.1	78.9	25
				epididymis_M2	11.4	15.8	28	72	17
				epididymis_M3	9.8	31.2	38	62	21
				epididymis_M4	13.2	25.6	34	66	20
				epididymis_M5	14	31	37.1	62.9	19
				epididymis_M6	16.6	23.2	28.9	71.1	16
				epididymis_M7	13.9	41.4	49	51	21
				epididymis_M8	10.6	25.8	36	64	22
				epididymis_M9	18.8	38.5	42	58	16
				epididymis_M10	10.4	38.5	44	56	17
				epididymis_M11	12.4	38.4	44	56	16
				epididymis_M12	11.8	45	55	45	17
**mean**					**12.8**	**30.5**	**38.09**	**61.91**	**18.9**
	6	*Cfap161*$^{\Delta ex2,3/\Delta ex2,3}$	3	epididymis_M1	37.8	86.2	89	11	16
				epididymis_M2	26	90	91	9	17
				epididymis_M3	38.4	68.5	71	29	15
				epididymis_M4	37.2	80.4	82.9	17.1	14
				epididymis_M5	32.8	66.7	74	26	16
				epididymis_M6	30.2	63.8	71	29	16
				epididymis_M7	19	15.1	25	75	18
				epididymis_M8	13.2	20.3	35	65	16
				epididymis_M9	14.6	28	31	69	16
				epididymis_M10	24.8	37.2	40	60	20
				epididymis_M11	16.6	41.7	52	48	14
				epididymis_M12	17.6	31.7	39	61	17
**mean**					**25.7**	**52.5**	**58.41**	**41.59**	**16.3**

Table S7. Gene-ontology analysis of significantly differentially expressed genes. Table includes 3 sheets with results of GO-analysis.

Sheet 1: Cfap161-DEgenes-GO-cellular component

Analysis Type:	PANTHER Overrepresentation Test (Released 20200728)
Annotation Version and Release Date:	GO Ontology database DOI: 10.5281/zenodo.3980761 Released 2020-08-10
Analyzed List:	upload_1 (Mus musculus)
Reference List:	Mus musculus (all genes in database)
Test Type:	FISHER
Correction:	FDR

GO cellular component complete	Mus musculus - REFLIST (22265)	upload_1 (276)	upload_1 (expected)	upload_1 (over/under)	upload_1 (fold Enrichment)	upload_1 (raw P-value)	upload_1 (FDR)
inner dynein arm (GO:0036156)	12	3	0,15	+	20,17	7,41E-04	3,95E-02
axonemal dynein complex (GO:0005858)	24	4	0,3	+	13,45	3,57E-04	2,43E-02
spindle midzone (GO:0051233)	36	5	0,45	+	11,2	1,39E-04	1,19E-02
dynein complex (GO:0030286)	55	6	0,68	+	8,8	1,01E-04	9,05E-03
condensed chromosome, centromeric region (GO:0000779)	57	5	0,71	+	7,08	9,72E-04	4,92E-02
SWI/SNF superfamily-type complex (GO:0070603)	74	6	0,92	+	6,54	4,49E-04	2,86E-02
ATPase complex (GO:1904949)	79	6	0,98	+	6,13	6,21E-04	3,51E-02
filopodium (GO:0030175)	93	7	1,15	+	6,07	2,32E-04	1,64E-02
histone acetyltransferase complex (GO:0000123)	80	6	0,99	+	6,05	6,61E-04	3,63E-02
microtubule associated complex (GO:0005875)	150	9	1,86	+	4,84	1,56E-04	1,28E-02
cytoplasmic region (GO:0099568)	220	10	2,73	+	3,67	5,70E-04	3,31E-02
centrosome (GO:0005813)	584	20	7,24	+	2,76	6,06E-05	5,99E-03
microtubule cytoskeleton (GO:0015630)	1203	40	14,91	+	2,68	2,20E-08	3,34E-06
ribonucleoprotein complex (GO:1990904)	700	23	8,68	+	2,65	3,16E-05	3,28E-03
nuclear body (GO:0016604)	683	21	8,47	+	2,48	1,71E-04	1,30E-02
cilium (GO:0005929)	674	20	8,35	+	2,39	5,49E-04	3,29E-02
cytoskeleton (GO:0005856)	2168	64	26,87	+	2,38	7,70E-11	3,04E-08

microtubule organizing center (GO:0005815)	717	21	8,89	+	2,36	4,19E-04	2,76E-02
catalytic complex (GO:1902494)	1361	37	16,87	+	2,19	1,19E-05	1,38E-03
nucleoplasm (GO:0005654)	3349	82	41,51	+	1,98	9,92E-10	3,26E-07
supramolecular complex (GO:0099080)	1215	29	15,06	+	1,93	8,15E-04	4,23E-02
non-membrane-bounded organelle (GO:0043228)	4538	105	56,25	+	1,87	2,06E-11	4,06E-08
intracellular non-membrane-bounded organelle (GO:0043232)	4520	104	56,03	+	1,86	4,94E-11	3,25E-08
nuclear lumen (GO:0031981)	4158	92	51,54	+	1,78	9,62E-09	1,58E-06
intracellular organelle lumen (GO:0070013)	4573	99	56,69	+	1,75	5,40E-09	1,33E-06
organelle lumen (GO:0043233)	4574	99	56,7	+	1,75	5,43E-09	1,19E-06
membrane-enclosed lumen (GO:0031974)	4574	99	56,7	+	1,75	5,43E-09	1,07E-06
cell projection (GO:0042995)	2548	54	31,59	+	1,71	9,04E-05	8,51E-03
plasma membrane bounded cell projection (GO:0120025)	2320	48	28,76	+	1,67	4,86E-04	3,00E-02
protein-containing complex (GO:0032991)	5338	110	66,17	+	1,66	6,70E-09	1,20E-06
nucleus (GO:0005634)	7104	130	88,06	+	1,48	2,35E-07	3,09E-05
organelle (GO:0043226)	12652	210	156,84	+	1,34	5,05E-11	2,50E-08
cytoplasm (GO:0005737)	11107	184	137,68	+	1,34	2,72E-08	3,84E-06
intracellular organelle (GO:0043229)	12309	202	152,58	+	1,32	1,58E-09	4,46E-07
intracellular (GO:0005622)	14060	226	174,29	+	1,3	2,18E-11	2,15E-08
membrane-bounded organelle (GO:0043227)	11399	181	141,3	+	1,28	1,98E-06	2,45E-04
intracellular membrane-bounded organelle (GO:0043231)	10627	168	131,73	+	1,28	1,53E-05	1,68E-03
intrinsic component of membrane (GO:0031224)	6054	48	75,05	-	0,64	1,70E-04	1,34E-02
integral component of membrane (GO:0016021)	5875	46	72,83	-	0,63	1,89E-04	1,38E-02

Sheet 2: Cfap161-DEgenes-GO-molecular function

Analysis Type:	PANTHER Overrepresentation Test (Released 20200728)
Annotation Version and Release Date:	GO Ontology database DOI: 10.5281/zenodo.3980761 Released 2020-08-10
Analyzed List:	upload_1 (Mus musculus)
Reference List:	Mus musculus (all genes in database)
Test Type:	FISHER
Correction:	FDR

GO molecular function complete	Mus musculus - REFLIST (22265)	upload_1 (276)	upload_1 (expected)	upload_1 (over/under)	upload_1 (fold Enrichment)	upload_1 (raw P-value)	upload_1 (FDR)
ATP-dependent microtubule motor activity, minus-end-directed (GO:0008569)	21	5	0,26	+	19,21	1,41E-05	8,21E-03
dynein light intermediate chain binding (GO:0051959)	31	7	0,38	+	18,22	3,49E-07	1,62E-03
dynein intermediate chain binding (GO:0045505)	34	5	0,42	+	11,86	1,09E-04	2,97E-02
ATP-dependent microtubule motor activity (GO:1990939)	37	5	0,46	+	10,9	1,56E-04	3,81E-02
RNA polymerase binding (GO:0070063)	62	6	0,77	+	7,81	1,85E-04	4,09E-02
motor activity (GO:0003774)	127	10	1,57	+	6,35	7,53E-06	5,83E-03
chromatin DNA binding (GO:0031490)	93	7	1,15	+	6,07	2,32E-04	4,31E-02
protein C-terminus binding (GO:0008022)	229	11	2,84	+	3,87	1,94E-04	3,92E-02
ATPase activity (GO:0016887)	406	19	5,03	+	3,78	1,41E-06	2,19E-03
histone binding (GO:0042393)	269	12	3,33	+	3,6	1,94E-04	4,09E-02
mRNA binding (GO:0003729)	301	13	3,73	+	3,48	1,43E-04	3,70E-02
chromatin binding (GO:0003682)	640	22	7,93	+	2,77	2,45E-05	1,03E-02
calcium ion binding (GO:0005509)	618	21	7,66	+	2,74	4,42E-05	1,28E-02
nucleoside-triphosphatase activity (GO:0017111)	749	25	9,28	+	2,69	1,10E-05	7,27E-03

pyrophosphatase activity (GO:0016462)	801	25	9,93	+	2,52	3,23E-05	1,25E-02
hydrolase activity, acting on acid anhydrides, in phosphorus-containing anhydrides (GO:0016818)	804	25	9,97	+	2,51	3,43E-05	1,23E-02
hydrolase activity, acting on acid anhydrides (GO:0016817)	804	25	9,97	+	2,51	3,43E-05	1,14E-02
RNA binding (GO:0003723)	1102	31	13,66	+	2,27	3,49E-05	1,08E-02
metal ion binding (GO:0046872)	3531	68	43,77	+	1,55	1,81E-04	4,21E-02
cation binding (GO:0043169)	3620	69	44,87	+	1,54	2,12E-04	4,11E-02
protein binding (GO:0005515)	9311	151	115,42	+	1,31	2,10E-05	9,75E-03
molecular transducer activity (GO:0060089)	2327	8	28,85	-	0,28	3,72E-06	4,32E-03
signaling receptor activity (GO:0038023)	2327	8	28,85	-	0,28	3,72E-06	3,45E-03
transmembrane signaling receptor activity (GO:0004888)	2131	5	26,42	-	0,19	4,10E-07	9,51E-04
olfactory receptor activity (GO:0004984)	1135	1	14,07	-	0,07	1,96E-05	1,01E-02

Sheet 3: Cfap161-DEgenes-GO-bological process

Analysis Type:	PANTHER Overrepresentation Test (Released 20200728)
Annotation Version and Release Date:	GO Ontology database DOI: 10.5281/zenodo.3980761 Released 2020-08-10
Analyzed List:	upload_1 (Mus musculus)
Reference List:	Mus musculus (all genes in database)
Test Type:	FISHER
Correction:	FDR

**GO biological process complete**	**Mus musculus - REFLIST (22265)**	**upload_1 (276)**	**upload_1 (expected)**	**upload_1 (over/under)**	**upload_1 (fold Enrichment)**	**upload_1 (raw P-value)**	**upload_1 (FDR)**
meiotic spindle organization (GO:0000212)	16	4	0,2	+	20,17	9,13E-05	4,24E-02
protein localization to cytoskeleton (GO:0044380)	45	6	0,56	+	10,76	3,62E-05	2,49E-02
mRNA stabilization (GO:0048255)	45	6	0,56	+	10,76	3,62E-05	2,38E-02
RNA stabilization (GO:0043489)	52	6	0,64	+	9,31	7,57E-05	3,99E-02
negative regulation of mRNA catabolic process (GO:1902373)	55	6	0,68	+	8,8	1,01E-04	4,55E-02
negative regulation of mRNA metabolic process (GO:1903312)	86	8	1,07	+	7,5	2,05E-05	1,62E-02
chromatin remodeling (GO:0006338)	149	13	1,85	+	7,04	1,07E-07	3,39E-04
microtubule bundle formation (GO:0001578)	107	8	1,33	+	6,03	8,72E-05	4,17E-02
regulation of mRNA processing (GO:0050684)	154	11	1,91	+	5,76	6,33E-06	7,14E-03
cilium movement (GO:0003341)	156	11	1,93	+	5,69	7,10E-06	7,48E-03
DNA packaging (GO:0006323)	153	10	1,9	+	5,27	3,42E-05	2,46E-02
regulation of RNA splicing (GO:0043484)	159	10	1,97	+	5,07	4,66E-05	2,83E-02
DNA conformation change (GO:0071103)	194	12	2,4	+	4,99	9,59E-06	9,47E-03
regulation of mRNA metabolic process (GO:1903311)	264	16	3,27	+	4,89	4,06E-07	5,83E-04

cytoskeleton-dependent intracellular transport (GO:0030705)	185	11	2,29	+	4,8	3,18E-05	2,39E-02
microtubule-based movement (GO:0007018)	352	19	4,36	+	4,35	1,81E-07	3,18E-04
regulation of transmembrane receptor protein serine/threonine kinase signaling pathway (GO:0090092)	245	12	3,04	+	3,95	8,40E-05	4,15E-02
microtubule cytoskeleton organization (GO:0000226)	506	23	6,27	+	3,67	1,75E-07	3,95E-04
chromatin organization (GO:0006325)	650	28	8,06	+	3,48	2,25E-08	1,19E-04
microtubule-based process (GO:0007017)	757	32	9,38	+	3,41	3,20E-09	2,53E-05
histone modification (GO:0016570)	334	14	4,14	+	3,38	1,08E-04	4,75E-02
organelle assembly (GO:0070925)	669	24	8,29	+	2,89	5,22E-06	6,87E-03
chromosome organization (GO:0051276)	943	33	11,69	+	2,82	1,39E-07	3,65E-04
cytoskeleton organization (GO:0007010)	1100	37	13,64	+	2,71	5,96E-08	2,35E-04
organelle organization (GO:0006996)	3145	81	38,99	+	2,08	9,99E-11	1,58E-06
movement of cell or subcellular component (GO:0006928)	1424	36	17,65	+	2,04	6,12E-05	3,45E-02
gene expression (GO:0010467)	1730	42	21,45	+	1,96	3,79E-05	2,39E-02
cellular component assembly (GO:0022607)	2017	46	25	+	1,84	8,14E-05	4,14E-02
cellular component biogenesis (GO:0044085)	2244	50	27,82	+	1,8	5,15E-05	3,01E-02
cellular component organization (GO:0016043)	5114	102	63,39	+	1,61	2,57E-07	4,06E-04
cellular component organization or biogenesis (GO:0071840)	5308	105	65,8	+	1,6	1,76E-07	3,48E-04
macromolecule metabolic process (GO:0043170)	5189	97	64,32	+	1,51	1,12E-05	9,85E-03
sensory perception (GO:0007600)	1642	5	20,35	-	0,25	6,28E-05	3,42E-02
G protein-coupled receptor signaling pathway (GO:0007186)	1829	5	22,67	-	0,22	1,10E-05	1,02E-02
sensory perception of smell (GO:0007608)	1123	1	13,92	-	0,07	1,90E-05	1,58E-02
sensory perception of chemical stimulus (GO:0007606)	1223	1	15,16	-	0,07	5,93E-06	7,21E-03

Table S8. Table includes 338 significantly differentially expressed genes (P-adj < 0.05).

Sort0	Gene name	Chr	wt1	wt 2	wt3	mut1	mut2	mut3	Base mean	log2(FC)	FC	StdErr	Wald-Stats	P-value	P-adj
1	Cfap161	chr7	3261	3134	3060	7506	7694	7664	5386	1,21	2,31	0,05	25,15	1,51E-139	0,0000
2	Gm44570	chr7	11	5	5	408	362	426	203	2,37	5,17	0,11	21,52	9,96E-103	0,0000
3	Rps15a-ps8	chr4	285	303	293	15	18	15	155	-1,93	0,26	0,11	-17,45	3,34E-68	0,0000
4	Efl1	chr7	3399	3501	3305	1486	1574	1632	2483	-1,03	0,49	0,06	-17,35	1,84E-67	0,0000
5	Man2a2	chr7	6773	7367	7606	3593	3360	3357	5343	-0,99	0,50	0,06	-16,83	1,53E-63	0,0000
6	4933402C06Rik	chr7	4	8	10	360	639	667	281	1,83	3,56	0,11	16,75	5,45E-63	0,0000
7	Gm44827	chr7	269	232	237	11	8	12	128	-1,75	0,30	0,11	-15,83	1,95E-56	0,0000
8	Slco3a1	chr7	1559	1400	1533	2855	2775	2732	2142	0,82	1,77	0,06	13,13	2,21E-39	0,0000
9	Fam83e	chr7	670	741	670	1740	2662	2877	1560	1,21	2,31	0,10	11,63	2,89E-31	0,0000
10	Gm6866	chr7	15	25	25	170	289	273	133	1,18	2,27	0,11	10,90	1,13E-27	0,0000
11	Trim12a	chr7	1	0	2	121	100	95	53	1,04	2,06	0,10	10,35	4,00E-25	0,0000
12	Rrm1	chr7	5834	5811	5906	3934	4255	4073	4969	-0,49	0,71	0,05	-9,83	8,36E-23	0,0000
13	Pop4	chr7	357	370	381	707	738	759	552	0,82	1,77	0,08	9,80	1,12E-22	0,0000
14	1700025L06Rik	chr7	43	40	39	149	264	250	131	0,94	1,92	0,11	8,59	8,39E-18	0,0000
15	Fchsd2	chr7	1622	1527	1543	976	1042	1017	1288	-0,56	0,68	0,07	-8,50	1,88E-17	0,0000
16	Gm45441	chr7	1724	1667	1446	2493	3214	3257	2300	0,71	1,64	0,09	8,00	1,24E-15	0,0000
17	Ibsp	chr5	1032	931	1074	21	9	4	512	-0,70	0,62	0,09	-7,87	3,48E-15	0,0000
18	Frzb	chr2	1424	1234	1352	372	596	649	938	-0,83	0,56	0,11	-7,76	8,19E-15	0,0000
19	Hddc3	chr7	336	325	328	153	162	145	241	-0,77	0,59	0,10	-7,58	3,42E-14	0,0000
20	Mesd	chr7	4151	4566	4330	3159	3212	3251	3778	-0,41	0,75	0,05	-7,50	6,20E-14	0,0000
21	4933405O20Rik	chr7	1409	1435	1413	2011	2001	2277	1758	0,50	1,41	0,07	7,38	1,54E-13	0,0000
22	Klk1b26	chr7	24	63	54	187	239	229	133	0,76	1,69	0,11	7,11	1,20E-12	0,0000
23	4933406J10Rik	chr7	29	25	23	111	97	109	66	0,75	1,68	0,11	6,94	3,90E-12	0,0000
24	Gdpd4	chr7	1478	1403	1311	1986	1956	2172	1718	0,48	1,39	0,07	6,91	4,86E-12	0,0000
25	Fus	chr7	5215	5288	5754	4233	3991	4039	4753	-0,38	0,77	0,06	-6,69	2,26E-11	0,0000
26	Syne2	chr12	11179	10962	11584	8101	8409	9132	9895	-0,37	0,77	0,06	-6,68	2,47E-11	0,0000

27	Ccdc114	chr7	2715	2805	2858	2008	1374	1447	2201	-0,60	0,66	0,09	-6,39	1,69E-10	0,0000
28	BC048644	chr8	1463	1549	1662	2168	2170	2514	1921	0,48	1,39	0,07	6,39	1,64E-10	0,0000
29	Fah	chr7	177	190	172	77	73	74	127	-0,70	0,62	0,11	-6,38	1,79E-10	0,0000
30	1700021F07Rik	chr2	3157	2990	3161	4046	3920	3894	3528	0,33	1,26	0,05	6,34	2,23E-10	0,0000
31	Safb2	chr17	1576	1608	1788	1154	1128	1210	1411	-0,45	0,73	0,07	-6,30	2,91E-10	0,0000
32	Plec	chr15	27469	30177	34259	22392	21046	22227	26262	-0,43	0,74	0,07	-6,26	3,77E-10	0,0000
33	Nlrp14	chr7	9026	9544	8922	11447	11441	12672	10509	0,35	1,27	0,06	6,20	5,77E-10	0,0000
34	Vwa5a	chr9	462	481	500	309	245	273	378	-0,60	0,66	0,10	-6,13	8,52E-10	0,0000
35	Hnrnpul2	chr19	1720	1808	1993	1373	1187	1261	1557	-0,46	0,73	0,08	-6,01	1,86E-09	0,0000
36	Spem1	chr11	11954	13078	12289	16248	16258	14943	14128	0,32	1,25	0,05	5,97	2,42E-09	0,0000
37	Cchcr1	chr17	15097	15149	16158	12992	12317	12488	14033	-0,28	0,82	0,05	-5,88	4,03E-09	0,0000
38	Gm10602	chr7	268	266	221	451	445	411	344	0,58	1,49	0,10	5,86	4,68E-09	0,0000
39	Gm33586	chr7	220	302	365	132	106	129	209	-0,64	0,64	0,11	-5,83	5,66E-09	0,0000
40	Hnrnpa0	chr13	3088	3423	3685	2622	2235	2236	2882	-0,45	0,73	0,08	-5,79	6,85E-09	0,0000
41	Gm31393	chr14	36	61	62	149	152	149	101	0,63	1,55	0,11	5,76	8,50E-09	0,0000
42	Gm45868	chr7	0	0	0	42	35	32	18	0,43	1,35	0,08	5,73	1,03E-08	0,0000
43	Hnrnpu	chr1	5872	6087	6392	5074	4510	4690	5437	-0,33	0,80	0,06	-5,64	1,72E-08	0,0000
44	Sec1	chr7	1464	1258	1226	1735	2238	2063	1664	0,49	1,40	0,09	5,60	2,14E-08	0,0000
45	Tex35	chr1	5275	5053	5174	6086	6597	6406	5765	0,28	1,21	0,05	5,60	2,19E-08	0,0000
46	4932431P20Rik	chr7	2044	1979	2170	1605	1536	1615	1825	-0,35	0,78	0,06	-5,52	3,41E-08	0,0000
47	Tmem232	chr17	4967	4538	4674	5692	5940	6202	5335	0,31	1,24	0,06	5,49	4,13E-08	0,0000
48	Dhx9	chr1	4403	4117	4772	3494	3355	3436	3930	-0,34	0,79	0,06	-5,45	5,18E-08	0,0000
49	Ngrn	chr7	580	668	649	441	403	399	523	-0,49	0,71	0,09	-5,45	5,10E-08	0,0000
50	Art1	chr7	121	110	86	203	222	257	167	0,60	1,52	0,11	5,45	5,14E-08	0,0000
51	C530008M17Rik	chr5	14761	14953	18341	12128	11085	11782	13842	-0,40	0,76	0,07	-5,41	6,45E-08	0,0001
52	Cep164	chr9	6198	6679	7336	5259	4789	5254	5919	-0,36	0,78	0,07	-5,39	7,24E-08	0,0001
53	Fscn3	chr6	8536	8432	8246	9653	10289	10334	9248	0,25	1,19	0,05	5,32	1,06E-07	0,0001
54	4930533N22Rik	chr7	33	68	70	146	167	181	111	0,56	1,47	0,11	5,26	1,47E-07	0,0001
55	Dnhd1	chr7	11461	11394	11803	9331	9905	9882	10629	-0,24	0,85	0,05	-5,25	1,56E-07	0,0001

56	Eif5b	chr1	7035	7046	7131	6134	5940	5941	6538	-0,23	0,85	0,04	-5,23	1,74E-07	0,0001
57	Mtcl1	chr17	1147	1131	1296	905	814	844	1023	-0,41	0,75	0,08	-5,21	1,89E-07	0,0001
58	Tssk6	chr8	19671	19226	18055	22396	22561	22891	20800	0,24	1,18	0,05	5,19	2,12E-07	0,0002
59	Taf15	chr11	1258	1404	1462	1015	994	1035	1195	-0,38	0,77	0,07	-5,15	2,57E-07	0,0002
60	Fam71e1	chr7	13102	13546	12406	15352	16145	15480	14339	0,25	1,19	0,05	5,13	2,95E-07	0,0002
61	Trpm4	chr7	2678	2512	2539	2154	1910	1930	2287	-0,33	0,80	0,07	-5,10	3,39E-07	0,0002
62	Myo7a	chr7	3654	3757	4430	5130	5225	5224	4570	0,35	1,27	0,07	5,09	3,54E-07	0,0002
63	Klk1b22	chr7	4	6	3	63	25	71	28	0,40	1,32	0,08	5,07	4,06E-07	0,0003
64	Ntn5	chr7	61	67	83	129	182	183	118	0,56	1,47	0,11	5,06	4,09E-07	0,0003
65	Syt9	chr7	306	253	238	132	156	139	204	-0,55	0,68	0,11	-5,07	4,07E-07	0,0003
66	Carm1	chr9	3959	3806	4011	3356	3122	3079	3555	-0,28	0,82	0,06	-5,03	4,95E-07	0,0003
67	Bc1	chr7	810	833	588	1475	1168	1108	997	0,51	1,42	0,10	5,02	5,05E-07	0,0003
68	Gm44748	chr7	0	3	0	23	65	47	23	0,35	1,27	0,07	4,99	6,06E-07	0,0004
69	Gm5592	chr7	50	37	67	114	173	136	96	0,53	1,44	0,11	4,97	6,62E-07	0,0004
70	Tex24	chr8	1547	1490	1491	1195	976	1142	1307	-0,39	0,76	0,08	-4,95	7,43E-07	0,0004
71	Rpgrip1	chr14	7817	7680	8321	6808	5916	6277	7136	-0,30	0,81	0,06	-4,91	8,98E-07	0,0005
72	Macf1	chr4	8415	9395	10059	7613	6490	7036	8168	-0,35	0,78	0,07	-4,90	9,39E-07	0,0005
73	Fat1	chr8	2634	2597	3120	2109	2115	2084	2443	-0,36	0,78	0,07	-4,90	9,64E-07	0,0005
74	Dnah14	chr1	18107	18288	18948	16076	16056	15976	17242	-0,20	0,87	0,04	-4,87	1,12E-06	0,0006
75	Pdzph1	chr17	625	629	643	816	859	877	742	0,37	1,29	0,08	4,87	1,11E-06	0,0006
76	1700046C09Rik	chr11	150	135	268	379	582	378	315	0,53	1,44	0,11	4,85	1,24E-06	0,0007
77	Snrnp70	chr7	2692	3051	3391	2420	1891	2041	2581	-0,42	0,75	0,09	-4,83	1,34E-06	0,0007
78	Acin1	chr14	7853	8003	8332	6987	6827	6751	7459	-0,22	0,86	0,05	-4,84	1,32E-06	0,0007
79	Dnajb8	chr6	9776	8925	8805	10853	11080	11269	10118	0,25	1,19	0,05	4,83	1,39E-06	0,0007
80	Hypm	chrX	2513	2783	2329	3179	3716	3308	2971	0,36	1,28	0,08	4,82	1,46E-06	0,0008
81	Ubqlnl	chr7	10391	9465	8909	11449	12118	12545	10813	0,30	1,23	0,06	4,80	1,55E-06	0,0008
82	Prm1	chr16	119563	132622	112827	149684	151404	148583	135780	0,28	1,21	0,06	4,80	1,56E-06	0,0008
83	1700027A15Rik	chr1	7959	7231	5741	9151	10528	10022	8439	0,41	1,33	0,09	4,80	1,59E-06	0,0008
84	Eif3a	chr19	6306	6361	6858	5372	5476	5466	5973	-0,24	0,85	0,05	-4,79	1,66E-06	0,0008

85	Traf3ip1	chr1	1886	1890	1952	1606	1424	1466	1704	-0,32	0,80	0,07	-4,79	1,68E-06	0,0008
86	Lrp1	chr10	3088	3422	4070	2697	2496	2422	3033	-0,39	0,76	0,08	-4,78	1,78E-06	0,0009
87	Actrt2	chr4	18276	19325	16886	21847	22158	21954	20074	0,26	1,20	0,05	4,77	1,87E-06	0,0009
88	Eif3k	chr7	4270	3911	4116	4800	4979	5104	4530	0,26	1,20	0,05	4,75	2,01E-06	0,0009
89	Catsperg1	chr7	4661	5020	5458	4228	3628	3694	4448	-0,34	0,79	0,07	-4,75	2,01E-06	0,0009
90	Msl1	chr11	11392	11412	11906	10202	9811	9708	10738	-0,21	0,86	0,05	-4,76	1,96E-06	0,0009
91	Tsga8	chrX	6403	6596	6000	7298	8166	8037	7083	0,28	1,21	0,06	4,76	1,97E-06	0,0009
92	Rbmxl2	chr7	3683	3839	3803	4569	4363	4850	4185	0,26	1,20	0,06	4,72	2,39E-06	0,0011
93	Gm31166	chr19	1616	1511	1670	1252	1100	1273	1404	-0,35	0,78	0,07	-4,72	2,39E-06	0,0011
94	Abl2	chr1	2115	2198	2352	1770	1800	1775	2002	-0,29	0,82	0,06	-4,71	2,50E-06	0,0011
95	Trim30d	chr7	20	7	3	77	56	76	40	0,38	1,30	0,08	4,67	2,96E-06	0,0013
96	Trim41	chr11	3067	3384	3802	2741	2424	2534	2992	-0,36	0,78	0,08	-4,66	3,23E-06	0,0014
97	Mdn1	chr4	9148	9633	10321	7251	7778	8288	8736	-0,29	0,82	0,06	-4,64	3,49E-06	0,0015
98	Chd5	chr4	15463	17292	18877	14193	13008	13317	15358	-0,31	0,81	0,07	-4,63	3,65E-06	0,0015
99	Abcc1	chr16	2033	2030	2283	1653	1691	1679	1895	-0,30	0,81	0,07	-4,62	3,93E-06	0,0016
100	Gpx4	chr10	23073	24988	22469	27551	28366	27653	25683	0,23	1,17	0,05	4,59	4,33E-06	0,0018
101	Neurl1a	chr19	1480	1459	1382	1798	1746	1800	1611	0,28	1,21	0,06	4,55	5,24E-06	0,0022
102	Gm6124	chr7	284	278	281	136	194	159	222	-0,49	0,71	0,11	-4,54	5,55E-06	0,0023
103	Scarna2	chr3	3228	3189	3182	3966	3639	3900	3517	0,24	1,18	0,05	4,52	6,26E-06	0,0025
104	Notch1	chr2	581	622	627	472	396	418	519	-0,40	0,76	0,09	-4,51	6,45E-06	0,0026
105	Abhd17a	chr10	4784	5064	4864	5766	5725	5667	5312	0,21	1,16	0,05	4,50	6,89E-06	0,0027
106	Gsg1	chr6	51041	52586	49822	57576	59476	57919	54737	0,18	1,13	0,04	4,48	7,37E-06	0,0029
107	P4ha3	chr7	26	48	56	5	8	7	25	-0,37	0,77	0,08	-4,48	7,33E-06	0,0029
108	Ccdc9	chr7	921	1027	1142	813	641	604	858	-0,43	0,74	0,10	-4,47	7,76E-06	0,0030
109	1810013L24Rik	chr16	6360	6299	7072	5530	5411	5398	6012	-0,25	0,84	0,06	-4,46	8,02E-06	0,0031
110	Ahnak	chr19	3546	3227	2978	2724	1867	1975	2720	-0,43	0,74	0,10	-4,44	8,98E-06	0,0034
111	Rabac1	chr7	2848	3096	2853	3689	3434	3556	3246	0,26	1,20	0,06	4,44	9,20E-06	0,0034
112	Cyb5r3	chr15	1170	1495	1660	1052	918	967	1210	-0,42	0,75	0,10	-4,43	9,32E-06	0,0035
113	Map4k4	chr1	1711	1800	2030	1492	1383	1393	1635	-0,33	0,80	0,07	-4,42	1,00E-05	0,0037

114	Phgdh-ps1	chr7	187	179	193	297	302	272	238	0,45	1,37	0,10	4,39	1,14E-05	0,0042
115	4930503B20Rik	chr3	6745	6454	6245	7459	7736	7534	7029	0,21	1,16	0,05	4,39	1,16E-05	0,0042
116	Safb	chr17	2051	2307	2432	1761	1843	1630	2004	-0,33	0,80	0,07	-4,38	1,21E-05	0,0043
117	Incenp	chr19	1643	1644	1762	1410	1252	1337	1508	-0,30	0,81	0,07	-4,36	1,30E-05	0,0046
118	Ubxn6	chr17	21993	22567	20648	24822	25291	25567	23481	0,20	1,15	0,05	4,35	1,37E-05	0,0048
119	Zfp609	chr9	2418	2596	2579	2120	2042	2127	2314	-0,25	0,84	0,06	-4,34	1,41E-05	0,0049
120	Cep350	chr1	17290	17631	18860	15271	15455	15596	16684	-0,20	0,87	0,05	-4,33	1,50E-05	0,0050
121	Aurka	chr2	5594	4962	5229	6064	6743	6477	5845	0,26	1,20	0,06	4,34	1,46E-05	0,0050
122	Arid1a	chr4	3904	4013	4381	3471	3287	3384	3740	-0,26	0,84	0,06	-4,33	1,50E-05	0,0050
123	Hnrnpa2b1	chr6	9761	9646	10189	8385	8750	8512	9207	-0,20	0,87	0,05	-4,33	1,47E-05	0,0050
124	Rps15a	chr7	2872	3093	2739	3659	3705	3377	3241	0,28	1,21	0,06	4,33	1,49E-05	0,0050
125	Zfc3h1	chr10	3388	3423	3608	2774	2781	3042	3170	-0,26	0,84	0,06	-4,33	1,52E-05	0,0051
126	Zbtb20	chr16	2409	2386	2445	1800	2098	1903	2174	-0,29	0,82	0,07	-4,32	1,54E-05	0,0051
127	Rrbp1	chr2	3079	3342	3501	2819	2620	2481	2974	-0,29	0,82	0,07	-4,31	1,61E-05	0,0053
128	1700067K01Rik	chr8	1136	1112	1181	1529	1361	1448	1294	0,30	1,23	0,07	4,31	1,64E-05	0,0053
129	AC163352.1	chr16	125	147	134	188	288	283	194	0,48	1,39	0,11	4,31	1,64E-05	0,0053
130	4930571K23Rik	chr7	23666	22943	21632	25813	27065	26499	24603	0,21	1,16	0,05	4,30	1,69E-05	0,0054
131	Cabs1	chr5	22812	22192	20385	24963	26661	25886	23817	0,23	1,17	0,05	4,29	1,78E-05	0,0056
132	Cstf1	chr2	4627	4000	4449	5050	5586	5671	4897	0,29	1,22	0,07	4,28	1,86E-05	0,0059
133	Serpinh1	chr7	464	499	519	371	288	341	414	-0,42	0,75	0,10	-4,26	2,01E-05	0,0062
134	Trim34a	chr7	21	17	18	58	58	53	38	0,42	1,34	0,10	4,26	2,02E-05	0,0062
135	Golga4	chr9	13369	14043	14688	11696	11814	12383	12999	-0,22	0,86	0,05	-4,26	2,01E-05	0,0062
136	Bicd2	chr13	1956	1918	2238	1631	1578	1619	1823	-0,30	0,81	0,07	-4,26	2,01E-05	0,0062
137	Gm6818	chr7	455	553	622	399	322	299	442	-0,45	0,73	0,10	-4,25	2,13E-05	0,0065
138	Ppp4c	chr7	3680	3948	3714	4430	4367	4724	4144	0,24	1,18	0,06	4,24	2,26E-05	0,0068
139	Ankfn1	chr11	120	147	138	194	248	294	190	0,47	1,39	0,11	4,23	2,32E-05	0,0069
140	Golgb1	chr16	4324	4299	4674	3649	3837	3704	4081	-0,23	0,85	0,05	-4,22	2,40E-05	0,0071
141	Dnah3	chr7	10528	10409	11311	8600	8416	9594	9810	-0,25	0,84	0,06	-4,21	2,61E-05	0,0077
142	Tmc5	chr7	5200	4901	4934	4323	4363	4231	4659	-0,21	0,86	0,05	-4,20	2,66E-05	0,0078

143	Ak3	chr19	954	869	936	1185	1270	1111	1054	0,32	1,25	0,08	4,19	2,76E-05	0,0080
144	Cenpe	chr3	4947	5084	5246	4407	4452	4188	4721	-0,21	0,86	0,05	-4,19	2,82E-05	0,0081
145	Nomo1	chr7	3411	3558	3674	3978	4591	4517	3955	0,27	1,21	0,06	4,18	2,88E-05	0,0083
146	Mrpl48	chr7	628	640	551	848	813	774	709	0,35	1,27	0,08	4,17	3,01E-05	0,0086
147	Zc3h13	chr14	647	671	697	526	504	485	588	-0,34	0,79	0,08	-4,17	3,06E-05	0,0086
148	1700019D03Rik	chr1	8014	8038	7621	8917	9254	9008	8475	0,19	1,14	0,05	4,17	3,08E-05	0,0087
149	4930432E11Rik	chr7	852	775	896	649	570	651	732	-0,35	0,78	0,09	-4,16	3,24E-05	0,0090
150	Plxdc1	chr11	2655	2565	2498	2957	3053	3183	2818	0,23	1,17	0,06	4,16	3,22E-05	0,0090
151	Dst	chr1	10914	11456	11559	9390	9479	10190	10498	-0,21	0,86	0,05	-4,15	3,34E-05	0,0092
152	Tanc2	chr11	2113	2020	1974	1604	1694	1729	1856	-0,26	0,84	0,06	-4,15	3,35E-05	0,0092
153	Hspg2	chr4	1248	1859	1987	1238	905	1052	1382	-0,44	0,74	0,11	-4,14	3,51E-05	0,0095
154	Hspb9	chr11	15119	16619	13376	19468	18114	18714	16902	0,28	1,21	0,07	4,14	3,52E-05	0,0095
155	Rpl31	chr1	2056	2231	2009	2628	2451	2591	2327	0,26	1,20	0,06	4,13	3,63E-05	0,0096
156	Dusp15	chr2	3904	3874	3714	4420	4550	5215	4280	0,27	1,21	0,07	4,13	3,64E-05	0,0096
157	1110017D15Rik	chr4	8337	9106	8458	10121	10273	9815	9352	0,21	1,16	0,05	4,13	3,59E-05	0,0096
158	Agpat2	chr2	4015	4280	4257	4919	5101	4747	4553	0,22	1,16	0,05	4,13	3,66E-05	0,0096
159	Bbs9	chr9	6767	6524	6487	7293	7994	7893	7160	0,22	1,16	0,05	4,12	3,71E-05	0,0097
160	1700042G07Rik	chr4	1923	2149	1928	2329	2535	2585	2242	0,28	1,21	0,07	4,12	3,79E-05	0,0098
161	Kmt2d	chr15	5206	5962	6306	4560	4912	4457	5234	-0,29	0,82	0,07	-4,12	3,81E-05	0,0098
162	Mad2l2	chr4	6008	5451	5242	6486	6702	6942	6139	0,25	1,19	0,06	4,12	3,86E-05	0,0099
163	Nell1	chr7	11	14	15	25	91	88	41	0,34	1,27	0,08	4,12	3,86E-05	0,0099
164	Notch2	chr3	1248	1323	1413	1033	1101	978	1183	-0,31	0,81	0,08	-4,11	3,90E-05	0,0099
165	Glg1	chr8	2475	2586	2656	2143	2104	2222	2364	-0,24	0,85	0,06	-4,09	4,24E-05	0,0107
166	Hydin	chr8	12530	13340	13732	11648	10934	11370	12259	-0,21	0,86	0,05	-4,09	4,29E-05	0,0107
167	1700110I07Rik	chr7	87	92	87	142	170	159	123	0,45	1,37	0,11	4,08	4,52E-05	0,0113
168	Lrp8	chr4	3567	3796	3868	3253	3143	2897	3421	-0,25	0,84	0,06	-4,08	4,57E-05	0,0113
169	Pde8a	chr7	11555	11169	10967	12729	12623	12755	11966	0,17	1,13	0,04	4,07	4,62E-05	0,0113
170	Hirip3	chr7	2324	2307	2369	2049	1682	1821	2092	-0,29	0,82	0,07	-4,07	4,65E-05	0,0113
171	Dand5	chr8	879	813	812	991	1208	1111	969	0,34	1,27	0,08	4,07	4,61E-05	0,0113

172	Tex44	chr1	3099	2962	2926	3530	3519	3440	3246	0,21	1,16	0,05	4,06	4,85E-05	0,0117
173	Gm45564	chr7	3	0	0	16	30	37	14	0,26	1,20	0,06	4,06	4,97E-05	0,0120
174	AI429214	chr8	1154	1213	1183	1335	1740	1731	1393	0,35	1,27	0,09	4,05	5,22E-05	0,0124
175	Neat1	chr19	2243	1977	2576	1791	1733	1589	1985	-0,34	0,79	0,08	-4,05	5,21E-05	0,0124
176	Gigyf2	chr1	4723	4767	5143	4149	4243	4067	4515	-0,22	0,86	0,05	-4,04	5,36E-05	0,0127
177	Zfp326	chr5	1030	1049	1063	839	833	856	945	-0,28	0,82	0,07	-4,04	5,39E-05	0,0127
178	Chd4	chr6	12355	12491	12903	10993	11272	11173	11864	-0,17	0,89	0,04	-4,04	5,43E-05	0,0127
179	Btbd1	chr7	10494	10986	10341	11915	12014	12379	11355	0,18	1,13	0,05	4,03	5,67E-05	0,0132
180	Yif1b	chr7	3927	4181	3637	4652	4602	4922	4320	0,25	1,19	0,06	4,02	5,91E-05	0,0137
181	Hnrnpa3	chr2	1978	2217	2154	1833	1618	1532	1889	-0,30	0,81	0,08	-4,01	5,98E-05	0,0137
182	1700120K04Rik	chr7	491	416	429	631	715	562	541	0,39	1,31	0,10	4,01	5,99E-05	0,0137
183	Oxct2a	chr4	4930	5071	5005	5584	5784	5756	5355	0,18	1,13	0,05	4,01	6,11E-05	0,0138
184	Ep300	chr15	4229	4150	4468	3621	3714	3693	3979	-0,21	0,86	0,05	-4,01	6,09E-05	0,0138
185	1700012A03Rik	chr6	6951	7078	6053	7896	8385	8060	7404	0,25	1,19	0,06	4,00	6,41E-05	0,0143
186	4930433I11Rik	chr7	910	930	878	1081	1195	1134	1021	0,29	1,22	0,07	4,00	6,42E-05	0,0143
187	Bptf	chr11	5720	5635	5970	5104	5035	4914	5396	-0,19	0,88	0,05	-4,00	6,41E-05	0,0143
188	Pabpn1	chr14	1214	1301	1343	1105	843	878	1114	-0,36	0,78	0,09	-3,99	6,58E-05	0,0146
189	Olfr654	chr7	5	0	0	36	24	29	16	0,26	1,20	0,06	3,99	6,75E-05	0,0148
190	Gm10039	chr11	53	35	36	51	209	207	99	0,35	1,27	0,09	3,98	6,79E-05	0,0148
191	Dync1h1	chr12	35243	41084	43588	32859	32537	32277	36265	-0,27	0,83	0,07	-3,98	6,76E-05	0,0148
192	Nucb1	chr7	1218	1182	1115	1425	1512	1402	1309	0,27	1,21	0,07	3,97	7,09E-05	0,0153
193	Adamts19	chr18	55	52	65	93	129	129	87	0,43	1,35	0,11	3,97	7,10E-05	0,0153
194	Cgn	chr3	1287	1359	1688	1154	1013	971	1245	-0,36	0,78	0,09	-3,96	7,43E-05	0,0159
195	Spen	chr4	6505	7031	7535	5982	5953	5508	6419	-0,25	0,84	0,06	-3,96	7,41E-05	0,0159
196	Khnyn	chr14	452	401	413	569	598	544	496	0,35	1,27	0,09	3,95	7,67E-05	0,0163
197	Crebbp	chr16	3877	4003	4171	3514	3452	3383	3733	-0,21	0,86	0,05	-3,95	7,91E-05	0,0167
198	Myt1	chr2	718	634	711	494	482	555	599	-0,35	0,78	0,09	-3,93	8,42E-05	0,0177
199	Zbtb10	chr3	2968	2988	3270	2564	2513	2653	2826	-0,23	0,85	0,06	-3,93	8,65E-05	0,0181
200	Npm3	chr19	2751	2538	2310	3143	3292	2987	2837	0,28	1,21	0,07	3,92	8,76E-05	0,0182

201	1700001O22Rik	chr2	7135	6766	6518	7716	8282	7840	7376	0,21	1,16	0,05	3,92	8,86E-05	0,0183
202	Ndufc2	chr7	916	1069	951	1315	1234	1201	1115	0,30	1,23	0,08	3,92	8,93E-05	0,0184
203	Bmpr2	chr1	1794	1589	1641	1393	1376	1301	1516	-0,27	0,83	0,07	-3,90	9,56E-05	0,0193
204	Sptan1	chr2	16025	16956	17697	14898	14639	14541	15793	-0,19	0,88	0,05	-3,90	9,49E-05	0,0193
205	Ttll1	chr15	912	796	753	1168	972	1262	977	0,36	1,28	0,09	3,90	9,55E-05	0,0193
206	2610318N02Rik	chr16	18682	19081	17977	20419	21345	21704	19868	0,18	1,13	0,05	3,90	9,54E-05	0,0193
207	Uvrag	chr7	634	587	551	787	736	793	681	0,32	1,25	0,08	3,89	9,86E-05	0,0198
208	Atp2a2	chr5	9339	9531	9863	8744	7879	7765	8854	-0,22	0,86	0,06	-3,89	1,01E-04	0,0202
209	Nf2	chr11	6980	7301	6690	6297	5439	5688	6399	-0,24	0,85	0,06	-3,89	1,02E-04	0,0202
210	Znrf4	chr17	22058	24361	21797	27863	25504	26999	24764	0,22	1,16	0,06	3,88	1,02E-04	0,0203
211	Cic	chr7	2753	2783	2960	2424	2405	2413	2623	-0,21	0,86	0,06	-3,88	1,03E-04	0,0204
212	1700011C11Rik	chr7	7	7	8	29	53	26	21	0,31	1,24	0,08	3,88	1,05E-04	0,0205
213	Chd8	chr14	3441	3525	3730	2964	3095	3076	3305	-0,21	0,86	0,05	-3,87	1,10E-04	0,0214
214	Gpx4-ps2	chr10	1347	1411	1351	1690	1714	1574	1514	0,25	1,19	0,06	3,87	1,11E-04	0,0214
215	Tpt1	chr14	7510	7887	7057	9043	8603	8593	8115	0,21	1,16	0,05	3,87	1,10E-04	0,0214
216	Cfap54	chr10	6453	6306	6608	5613	5788	5635	6067	-0,18	0,88	0,05	-3,86	1,13E-04	0,0218
217	Gnb2	chr5	4477	4353	4253	5057	5003	4905	4675	0,18	1,13	0,05	3,86	1,15E-04	0,0220
218	Dnah10	chr5	6799	6878	7440	5821	6033	6230	6534	-0,21	0,86	0,05	-3,86	1,16E-04	0,0221
219	2410015M20Rik	chr17	4780	5237	4594	5655	5752	5887	5318	0,22	1,16	0,06	3,85	1,17E-04	0,0222
220	Srsf5	chr12	4137	4449	4680	3709	3678	3825	4080	-0,22	0,86	0,06	-3,85	1,20E-04	0,0227
221	Ltbp4	chr7	1170	1258	1444	1081	913	851	1120	-0,35	0,78	0,09	-3,84	1,23E-04	0,0232
222	Gm35360	chr14	28	30	31	72	61	75	50	0,40	1,32	0,10	3,84	1,25E-04	0,0234
223	Ubr4	chr4	35120	36244	39773	29965	31896	32563	34260	-0,22	0,86	0,06	-3,83	1,28E-04	0,0237
224	Trrap	chr5	7333	8449	9211	6660	6512	6959	7521	-0,27	0,83	0,07	-3,83	1,28E-04	0,0237
225	Pldi	chr10	543	613	581	756	714	789	666	0,32	1,25	0,08	3,83	1,28E-04	0,0237
226	Thap7	chr16	1581	1555	1528	1895	1923	1769	1709	0,24	1,18	0,06	3,82	1,31E-04	0,0241
227	Gm6155	chr7	22	28	25	71	58	57	44	0,39	1,31	0,10	3,82	1,32E-04	0,0241
228	Tmem217	chr17	705	418	371	899	823	809	671	0,42	1,34	0,11	3,82	1,32E-04	0,0241
229	Prrc2c	chr1	5414	5654	6231	4746	4987	4799	5305	-0,23	0,85	0,06	-3,81	1,37E-04	0,0247

230	2610206C17Rik	chr7	67	47	50	102	107	119	82	0,42	1,34	0,11	3,81	1,37E-04	0,0247
231	Lyrm7	chr11	1295	1136	1087	1544	1878	1392	1388	0,35	1,27	0,09	3,81	1,40E-04	0,0252
232	Fam186a	chr15	3150	3150	3219	2718	2577	2829	2941	-0,21	0,86	0,06	-3,81	1,41E-04	0,0252
233	Gm43352	chr3	1172	1278	1258	1497	1540	1464	1368	0,25	1,19	0,07	3,80	1,42E-04	0,0253
234	Slfnl1	chr4	13229	12571	12739	14169	14657	14522	13648	0,16	1,12	0,04	3,80	1,45E-04	0,0256
235	Rsf1	chr7	2591	2503	2555	2133	2170	2247	2366	-0,21	0,86	0,05	-3,80	1,44E-04	0,0256
236	Ttc9	chr12	1472	1266	1348	1772	1674	1627	1526	0,27	1,21	0,07	3,80	1,45E-04	0,0256
237	Igfbp5	chr1	358	302	319	219	91	180	245	-0,41	0,75	0,11	-3,79	1,48E-04	0,0260
238	Rps15	chr10	3623	3920	3488	4216	4843	4350	4073	0,25	1,19	0,07	3,79	1,52E-04	0,0265
239	Ppp1r9b	chr11	1544	1638	1742	1419	1283	1249	1479	-0,28	0,82	0,07	-3,79	1,53E-04	0,0265
240	Txndc2	chr17	15825	16413	15349	17445	19455	18305	17132	0,20	1,15	0,05	3,79	1,53E-04	0,0265
241	Soga1	chr2	997	903	1166	720	732	818	889	-0,34	0,79	0,09	-3,77	1,61E-04	0,0278
242	Fam78a	chr2	9973	9897	9854	10882	11199	11026	10472	0,15	1,11	0,04	3,77	1,67E-04	0,0286
243	Tpbgl	chr7	807	887	923	701	689	653	777	-0,30	0,81	0,08	-3,76	1,69E-04	0,0290
244	Zfp27	chr7	64	93	67	101	221	185	122	0,39	1,31	0,10	3,76	1,71E-04	0,0292
245	Arhgap17	chr7	692	737	757	503	604	547	640	-0,33	0,80	0,09	-3,76	1,73E-04	0,0293
246	Nxt1	chr2	3664	3669	3295	4089	4216	4203	3856	0,22	1,16	0,06	3,75	1,78E-04	0,0294
247	Josd2	chr7	1009	1024	971	1168	1415	1243	1138	0,30	1,23	0,08	3,75	1,80E-04	0,0294
248	A030001D20Rik	chr7	9	12	6	25	48	42	24	0,31	1,24	0,08	3,75	1,78E-04	0,0294
249	4933439N14Rik	chr8	223	161	169	302	271	292	237	0,40	1,32	0,11	3,75	1,80E-04	0,0294
250	Cep131	chr11	1419	1624	1583	1326	960	1124	1339	-0,34	0,79	0,09	-3,75	1,77E-04	0,0294
251	Aspscr1	chr11	7604	7589	7528	8686	8348	8441	8033	0,16	1,12	0,04	3,75	1,79E-04	0,0294
252	4930452G13Rik	chr14	1427	1354	1271	1715	1765	1549	1513	0,27	1,21	0,07	3,75	1,76E-04	0,0294
253	Spatc1	chr15	4854	5060	4626	5570	5615	5545	5212	0,19	1,14	0,05	3,75	1,78E-04	0,0294
254	Pgp	chr17	35472	35725	32606	39931	38551	43342	37605	0,21	1,16	0,06	3,75	1,77E-04	0,0294
255	Atrx	chrX	1157	1107	1210	919	909	980	1047	-0,27	0,83	0,07	-3,75	1,76E-04	0,0294
256	Numa1	chr7	2480	2934	2981	2408	2115	2105	2504	-0,29	0,82	0,08	-3,74	1,82E-04	0,0295
257	Baz2a	chr10	16289	15985	17205	14012	14561	14846	15483	-0,18	0,88	0,05	-3,74	1,84E-04	0,0298
258	Ovol2	chr2	1726	1912	1879	2476	2138	2193	2054	0,27	1,21	0,07	3,73	1,91E-04	0,0306

259	Pold3	chr7	4721	4904	4768	5370	5435	5470	5112	0,17	1,13	0,05	3,73	1,90E-04	0,0306
260	Igf2r	chr17	1108	1169	1315	990	884	935	1067	-0,30	0,81	0,08	-3,73	1,91E-04	0,0306
261	Tnp1	chr1	38288	46374	36341	49851	52266	48467	45264	0,28	1,21	0,07	3,73	1,92E-04	0,0306
262	Prss58	chr6	2239	2369	2219	2561	2763	2734	2481	0,22	1,16	0,06	3,72	1,96E-04	0,0311
263	Huwe1	chrX	6520	6696	7436	5395	5977	5879	6317	-0,24	0,85	0,06	-3,72	1,97E-04	0,0311
264	Cib1	chr7	4743	4800	4282	5373	5371	5411	4997	0,21	1,16	0,06	3,72	2,00E-04	0,0315
265	Cdc73	chr1	3632	3534	3746	2997	3215	3174	3383	-0,20	0,87	0,05	-3,72	2,03E-04	0,0318
266	Tjp1	chr7	5245	5032	5413	4510	4668	4487	4892	-0,19	0,88	0,05	-3,71	2,05E-04	0,0320
267	Ech1	chr7	1672	1854	1483	1952	2319	2201	1913	0,31	1,24	0,08	3,71	2,06E-04	0,0322
268	Bod1l	chr5	2914	2963	3210	2534	2644	2528	2799	-0,22	0,86	0,06	-3,71	2,07E-04	0,0322
269	Ccdc96	chr5	5224	5027	5295	4643	4498	4156	4807	-0,21	0,86	0,06	-3,71	2,09E-04	0,0322
270	Clip1	chr5	11407	10941	11349	10234	9711	9953	10599	-0,17	0,89	0,04	-3,71	2,11E-04	0,0322
271	Rnf133	chr6	8454	7981	7597	8906	9903	9440	8714	0,22	1,16	0,06	3,71	2,09E-04	0,0322
272	Gm5617	chr9	22233	26000	22382	29860	26369	29619	26077	0,25	1,19	0,07	3,70	2,12E-04	0,0322
273	Klhl28	chr12	1965	1942	1905	1506	1674	1664	1776	-0,24	0,85	0,06	-3,71	2,11E-04	0,0322
274	Lemd3	chr10	1638	1705	1624	1410	1341	1417	1522	-0,23	0,85	0,06	-3,70	2,15E-04	0,0327
275	Polr2a	chr11	7832	8283	8815	7084	7302	7107	7737	-0,20	0,87	0,05	-3,69	2,22E-04	0,0336
276	Gm45159	chr7	59	42	58	15	20	22	36	-0,35	0,78	0,10	-3,69	2,26E-04	0,0340
277	Acaca	chr11	2594	2597	2673	2152	2222	2342	2430	-0,21	0,86	0,06	-3,68	2,30E-04	0,0346
278	Gpr160	chr3	6458	6404	6313	7006	7233	7500	6819	0,17	1,13	0,05	3,68	2,33E-04	0,0347
279	Lrrc37a	chr11	7748	7385	8190	6477	6436	7012	7208	-0,21	0,86	0,06	-3,68	2,33E-04	0,0347
280	Gm381	chrX	804	712	663	858	1027	1014	846	0,33	1,26	0,09	3,68	2,33E-04	0,0347
281	Ccdc88c	chr12	5244	6228	6532	5221	4480	4521	5371	-0,29	0,82	0,08	-3,68	2,34E-04	0,0347
282	Rpl18a	chr8	2417	2569	2523	2959	2996	2808	2712	0,21	1,16	0,06	3,67	2,40E-04	0,0353
283	Bmper	chr9	92	110	127	209	169	178	148	0,40	1,32	0,11	3,67	2,47E-04	0,0362
284	Spz1	chr13	38861	37745	35290	41445	43828	42532	39950	0,18	1,13	0,05	3,67	2,47E-04	0,0362
285	Cox7b2	chr5	3971	4415	3861	4929	4695	4864	4456	0,22	1,16	0,06	3,66	2,49E-04	0,0362
286	4930407I10Rik	chr15	9255	9747	10203	8720	8350	8353	9105	-0,19	0,88	0,05	-3,66	2,48E-04	0,0362
287	Irgc1	chr7	47528	48479	49255	54384	52308	55706	51277	0,15	1,11	0,04	3,66	2,51E-04	0,0364

288	Ncl	chr1	2929	2927	3140	2622	2594	2473	2781	-0,21	0,86	0,06	-3,66	2,55E-04	0,0365
289	4930401C15Rik	chr10	6	2	3	10	50	165	39	0,22	1,16	0,06	3,66	2,54E-04	0,0365
290	Fam8a1	chr13	4614	4894	4458	5580	5433	5208	5031	0,20	1,15	0,06	3,66	2,54E-04	0,0365
291	4930455C13Rik	chr10	593	535	540	698	704	732	634	0,30	1,23	0,08	3,65	2,60E-04	0,0372
292	Fuz	chr7	694	751	678	848	893	1034	816	0,31	1,24	0,09	3,65	2,61E-04	0,0372
293	2210011C24Rik	chr8	163	63	83	237	284	182	169	0,38	1,30	0,10	3,65	2,66E-04	0,0377
294	Pcdh17	chr14	203	146	152	257	420	241	237	0,40	1,32	0,11	3,65	2,67E-04	0,0377
295	Rps14	chr18	6964	8041	7394	8840	8503	8987	8122	0,22	1,16	0,06	3,64	2,72E-04	0,0384
296	Acbd3	chr1	2575	2433	2556	2228	2151	2091	2339	-0,21	0,86	0,06	-3,64	2,76E-04	0,0387
297	Gm12260	chr11	7039	8308	7661	9112	8945	9140	8368	0,22	1,16	0,06	3,64	2,76E-04	0,0387
298	Spred3	chr7	1555	1715	1852	1448	1220	1360	1525	-0,29	0,82	0,08	-3,63	2,80E-04	0,0391
299	1700101O22Rik	chr12	1287	1529	1446	1657	1887	1787	1599	0,28	1,21	0,08	3,62	2,95E-04	0,0410
300	Kdm3b	chr18	1806	1759	1912	1547	1573	1462	1676	-0,23	0,85	0,06	-3,62	2,95E-04	0,0410
301	Morf4l1	chr9	12770	11729	11313	13311	14150	14226	12917	0,20	1,15	0,06	3,62	2,97E-04	0,0410
302	Spert	chr14	17974	17618	17206	18917	21158	20625	18916	0,19	1,14	0,05	3,62	2,98E-04	0,0410
303	Sart1	chr19	1434	1537	1610	1334	1202	1149	1377	-0,27	0,83	0,08	-3,62	2,99E-04	0,0410
304	Myh9	chr15	909	941	1025	839	546	560	803	-0,38	0,77	0,10	-3,62	3,00E-04	0,0411
305	1700003G18Rik	chr7	575	733	621	401	529	399	543	-0,37	0,77	0,10	-3,61	3,02E-04	0,0411
306	Mrpl57	chr14	1709	1917	1650	2191	2097	2107	1945	0,25	1,19	0,07	3,61	3,03E-04	0,0412
307	Dicer1	chr12	1468	1439	1432	1146	1253	1216	1326	-0,24	0,85	0,07	-3,61	3,04E-04	0,0412
308	St8sia1	chr6	339	455	495	312	175	262	340	-0,40	0,76	0,11	-3,61	3,06E-04	0,0413
309	Bcas1	chr2	382	400	405	524	524	502	456	0,31	1,24	0,09	3,61	3,11E-04	0,0414
310	Gstm5	chr3	65629	65976	59864	69771	75319	78617	69196	0,21	1,16	0,06	3,61	3,10E-04	0,0414
311	Baz1b	chr5	1845	1841	1826	1570	1517	1608	1701	-0,21	0,86	0,06	-3,61	3,12E-04	0,0414
312	Gab2	chr7	1909	1945	2037	2318	2244	2315	2128	0,21	1,16	0,06	3,61	3,12E-04	0,0414
313	Herpud1	chr8	7330	7346	7208	8255	8385	7943	7745	0,16	1,12	0,04	3,61	3,09E-04	0,0414
314	Gm11224	chr4	86	102	123	63	48	42	77	-0,39	0,76	0,11	-3,60	3,13E-04	0,0414
315	Gm5901	chr7	1501	1369	1363	1637	1716	1738	1554	0,24	1,18	0,07	3,60	3,16E-04	0,0418
316	Gm23388	chr8	160	229	272	123	133	122	173	-0,40	0,76	0,11	-3,60	3,18E-04	0,0418

317	3110070M22Rik	chr13	2214	2261	2152	2541	2605	2521	2382	0,20	1,15	0,05	3,60	3,18E-04	0,0418
318	1700003E16Rik	chr6	19892	19137	18426	21338	21705	21423	20320	0,16	1,12	0,04	3,59	3,34E-04	0,0436
319	Heg1	chr16	2732	2402	3092	2216	1930	2249	2437	-0,30	0,81	0,08	-3,59	3,34E-04	0,0436
320	Ubr5	chr15	41367	42086	43646	37750	37136	38966	40158	-0,15	0,90	0,04	-3,58	3,38E-04	0,0439
321	Tada2b	chr5	2264	2310	2445	2088	1903	1916	2154	-0,23	0,85	0,06	-3,58	3,41E-04	0,0442
322	0610009L18Rik	chr11	2133	2099	1934	2464	2511	2336	2246	0,22	1,16	0,06	3,58	3,42E-04	0,0442
323	AC122375.2	chr16	2345	2167	2047	2526	2612	2698	2399	0,23	1,17	0,06	3,58	3,48E-04	0,0448
324	Hectd1	chr12	6687	7124	7387	6263	5992	6184	6606	-0,19	0,88	0,05	-3,58	3,49E-04	0,0449
325	Efhd1	chr1	13398	13401	13715	14892	14899	14883	14198	0,14	1,10	0,04	3,57	3,52E-04	0,0450
326	Itpr2	chr6	1070	1322	1375	1001	928	960	1109	-0,31	0,81	0,09	-3,57	3,58E-04	0,0457
327	Tmem202	chr9	1345	1355	1256	1526	1632	1576	1448	0,23	1,17	0,07	3,56	3,65E-04	0,0464
328	C130074G19Rik	chr1	547	587	647	493	366	290	489	-0,38	0,77	0,11	-3,56	3,68E-04	0,0466
329	Myo5b	chr18	3390	3139	3549	2848	2561	2937	3071	-0,24	0,85	0,07	-3,56	3,71E-04	0,0469
330	Entr1	chr2	13501	13410	12922	14402	15049	15217	14083	0,16	1,12	0,04	3,55	3,79E-04	0,0478
331	Fam71f1	chr6	8624	8498	7860	9078	10394	9952	9068	0,22	1,16	0,06	3,54	3,96E-04	0,0487
332	4930542C12Rik	chr9	696	528	527	804	786	791	689	0,34	1,27	0,09	3,54	3,95E-04	0,0487
333	Ranbp2	chr10	13264	12691	12983	10909	11386	11963	12199	-0,18	0,88	0,05	-3,55	3,91E-04	0,0487
334	Med13	chr11	6934	6589	7185	5922	6147	6085	6477	-0,18	0,88	0,05	-3,55	3,88E-04	0,0487
335	Helz	chr11	4049	4030	4439	3449	3695	3570	3872	-0,21	0,86	0,06	-3,55	3,92E-04	0,0487
336	Macrod1	chr19	630	543	579	724	746	766	665	0,29	1,22	0,08	3,54	3,95E-04	0,0487
337	Ms4a13	chr19	4577	4421	4200	4916	5049	5259	4737	0,19	1,14	0,05	3,55	3,90E-04	0,0487
338	Rrp12	chr19	2924	2860	2925	2316	2540	2577	2690	-0,21	0,86	0,06	-3,54	3,96E-04	0,0487

SUPPLEMENTAL MATERIAL AND METHODS

Localisation of the epitope recognised by α–pI:

The epitope recognised by the monoclonal α–pI antibody was localised to exon 1 by peptide blocking. α–pI was generated using the peptide `RMGNWNEDVYLEEERM` which is mainly coded by exon1 (`RMGNWNEDVYLEE`). Testis lysates of wild type and *Cfap161*$^{\Delta ex2,3/\Delta ex2,3}$ mice were analysed by Western blots using unblocked α–pI (50 ng/ml 5% milk in PBS/0.1% Tween20), α–pI blocked with 5 µg `RMGNWNEDVYLEE` peptide (RMG-blocked) and α–pI blocked with 5 µg `ERMRHFLEKR` (ERM-blocked) for 1 hour at 37°C on a rotating wheel followed by centrifugation 15 min 10000 rpm.

Yeast two-hybrid (Y2H) assay to validate CFAP161-KIAA0556 interaction:

Yeast two-hybrid assays were performed according to the manufacturer's protocol (Matchmaker Gold Yeast Two-Hybrid System 630489, Clontech). Bait constructs (CFAP161, p53, Lam) were transformed into the Y2HGold yeast strain, prey constructs (KIAA0556 and large T) into the Y187 yeast strain using the Yeastmaker Yeast Transformation System 2. Co-expression of transformed plasmids was tested on double drop-out plates lacking leucin and tryptophan (DDO, SD/-Leu/-Trp). Interaction of bait and prey was investigated on quadruple drop-out plates lacking adenin, histidine, leucine and tryptophan (QDO, SD/-Ade/-His/-Leu/-Trp) supplemented with X-α-Gal (final 40 µg/ml) and Aureobasidin A (final 200 ng/ml). Plates were incubated at 30°C.

Computer assisted sperm analysis (CASA):

CASA was performed on 3 adult wild type and 3 *Cfap161*$^{\Delta ex2,3/\Delta ex2,3}$ (3-4 month aged) individuals as described [1]. Statistical analysis was performed with Prism7 (GraphPad) using paired t-test.

Transmission electron microscopy (TEM):

Cauda epididymides and lungs were dissected from 3 months old wild type and *Cfap161*$^{\Delta ex2,3/\Delta ex2,3}$ perfused litter mates (n=2), fixed, embedded and analysed as described in [2]. Animals were perfused with fixative through the portal vein. Used fixative was 1,5% glutaraldehyde/1,5% PFA in 0,15 M HEPES, pH 7.35.

RNA isolation for RNA-seq:

Total RNA was isolated from single testis without epididymis from 3 different 11-17 weeks old wild type and 3 different 11-13 weeks *Cfap161*$^{\Delta ex2,3/\Delta ex2,3}$ males using the Direct-zol RNA MiniPrep Kit (ZYMO) according to manufacturer's instructions. RNA integrity was first checked by running a gel. Then quality and amount was checked by Eukaryote Total RNA Nano and Bioanalyser.

RNA-seq:

Library generation, quality control, and quantification:

rRNA was depleted from 400ng of total RNA per sample with 'NEBNext rRNA Depletion Kit (Human/Mouse/Rat), 96 rxns' (E6310X; New England Biolabs) followed by stranded cDNA library generation using 'NEBNext Ultra II Directional RNA Library Prep Kit for Illumina' (E7760L; New England Biolabs). All reactions were performed according to user manualE7760 (Version 1.0_02-2017; NEB) but downscaled to 2/3 of initial volumes. One additional purification step was introduced at the end of the standard procedure, using 1x 'Agencourt AMPure XP Beads' (#A63881; Beckman Coulter, Inc.).

cDNA libraries were barcoded by single indexing approach, using 'NEBNext Multiplex Oligos for Illumina – Set 1' (Index Primer 2, 4, 5, 6, 7, 12). All generated cDNA libraries were amplified by 8 cycles of final PCR.

Fragment length distribution of individual libraries was monitored using 'Bioanalyzer High Sensitivity DNA Assay' (5067-4626; Agilent Technologies). Quantification of libraries was performed by use of the 'Qubit dsDNA HS Assay Kit' (Q32854; ThermoFisher Scientific).

Library denaturation and sequencing run:

Equal molar amounts of six individually barcoded libraries were pooled. Accordingly, each analysed library constitutes 16.6% of overall flowcell/run capacity. The library pool was denatured with NaOH and was finally diluted to 1.8 pM according to the Denature and Dilute Libraries Guide (Document # 15048776 v02; Illumina). 1.3 ml of denatured pool were loaded on an Illumina NextSeq 550 sequencer using a High Output Flowcell for 75bp single reads (#FC-404-2005; Illumina) with the following settings: Sequence read 1 with 76 bases; Index read 1 with 6 bases. On average 70.9 million reads were generated for each sample.

BCL to FASTQ conversion:

BCL files were converted to FASTQ files using bcl2fastq Conversion Software version v2.20.0.422 (Illumina).

Raw data processing and quality control:

Raw data were processed by nfcore/rnaseq (version 1.3), a bioinformatics best-practice analysis pipeline used for RNA sequencing data at the National Genomics Infrastructure at SciLifeLab Stockholm, Sweden. The genome reference and annotation data were taken from GENCODE.org (Mus musculus; GRCm38; release M18).

Normalisation and differential expression analysis:

DESeq2 (Galaxy Tool Version 2.11.40.2) was applied with default settings except for "Output normalised counts table", "Turn off outliers filtering", and "Turn off independent filtering", all of which were set to "True".

References

1. Beckers, A. *et al.* The FOXJ1 target Cfap206 is required for sperm motility, mucociliary clearance of the airways and brain development. *Development* **147,** (2020).

2. Rudat, C. *et al.* Upk3b is dispensable for development and integrity of urothelium and mesothelium. *PLoS ONE* **9,** e112112 (2014).
